# Transcriptomic stratification of late-onset Alzheimer's cases reveals novel genetic modifiers of disease pathology

**DOI:** 10.1371/journal.pgen.1008775

**Published:** 2020-06-03

**Authors:** Nikhil Milind, Christoph Preuss, Annat Haber, Guruprasad Ananda, Shubhabrata Mukherjee, Cai John, Sarah Shapley, Benjamin A. Logsdon, Paul K. Crane, Gregory W. Carter

**Affiliations:** 1 The Jackson Laboratory, Bar Harbor, Maine, United States of America; 2 Program in Genetics, Department of Biological Sciences, North Carolina State University, Raleigh, North Carolina, United States of America; 3 Department of Medicine, School of Medicine, University of Washington, Seattle, Washington, United States of America; 4 Program in Neuroscience, Department of Biology and Geology, Baldwin Wallace University, Berea, Ohio, United States of America; 5 Sage Bionetworks, Seattle, Washington, United States of America; HudsonAlpha Institute for Biotechnology, UNITED STATES

## Abstract

Late-Onset Alzheimer’s disease (LOAD) is a common, complex genetic disorder well-known for its heterogeneous pathology. The genetic heterogeneity underlying common, complex diseases poses a major challenge for targeted therapies and the identification of novel disease-associated variants. Case-control approaches are often limited to examining a specific outcome in a group of heterogenous patients with different clinical characteristics. Here, we developed a novel approach to define relevant transcriptomic endophenotypes and stratify decedents based on molecular profiles in three independent human LOAD cohorts. By integrating post-mortem brain gene co-expression data from 2114 human samples with LOAD, we developed a novel quantitative, composite phenotype that can better account for the heterogeneity in genetic architecture underlying the disease. We used iterative weighted gene co-expression network analysis (WGCNA) to reduce data dimensionality and to isolate gene sets that are highly co-expressed within disease subtypes and represent specific molecular pathways. We then performed single variant association testing using whole genome-sequencing data for the novel composite phenotype in order to identify genetic loci that contribute to disease heterogeneity. Distinct LOAD subtypes were identified for all three study cohorts (two in ROSMAP, three in Mayo Clinic, and two in Mount Sinai Brain Bank). Single variant association analysis identified a genome-wide significant variant in *TMEM106B* (p-value < 5×10^−8^, rs1990620^G^) in the ROSMAP cohort that confers protection from the inflammatory LOAD subtype. Taken together, our novel approach can be used to stratify LOAD into distinct molecular subtypes based on affected disease pathways.

## Author summary

One of the challenges of identifying risk variants for late-onset Alzheimer’s disease based on transcript abundance from post-mortem brain tissue is the inherent heterogeneity of human cohorts. This is evident from studies that have employed increasingly larger cohorts without detecting any new risk variants. In this study, we have developed a novel methodology to decompose transcriptomic data into submodules to reduce experimental noise and increase power by accounting for patient heterogeneity. We demonstrate the effectiveness of our submodules by identifying associated variants that are either novel or have been implicated previously in late-onset Alzheimer’s disease. A systems-level understanding of the transcriptome is especially important for translating human disease-associated variants into models that can accelerate the development of therapies.

## Introduction

Late-onset Alzheimer’s disease (LOAD) is the most common form of dementia in the elderly. The clinical features associated with LOAD are an amnesic type of memory impairment, deterioration of language, and visuospatial deficits. In the later stages of the disease, symptoms may include motor and sensory abnormalities, gait disturbances, and seizures. Without advances in therapy, the number of symptomatic cases in the United States is predicted to rise to 13.2 million by 2050 [1].

Many common, complex diseases such as LOAD present with heterogeneous phenotypes due to interactions between genetic and environmental factors affecting a range of pathways and processes. LOAD has no simple form of inheritance and is governed by a common set of risk alleles across multiple genes that, in combination, have a substantial effect on disease predisposition and age of onset [2]. Genome-Wide Association Studies (GWAS) have become an important tool for identifying variants in complex diseases [3,4]. GWAS for LOAD have identified variants in over 500 genes as potential risk factors with the ε4 variant in *APOE* as the strongest contributor to overall disease risk [2,5]. LOAD has a strong polygenic component and an estimated heritability of up to 80% [6]. It has been challenging to transition from the identification of associated genetic variants to the molecular mechanisms that lead to the accumulation of amyloid plaques and helical tau filaments [7]. Furthermore, there is mounting evidence that the observed heterogeneity in LOAD is associated with multiple distinct subtypes [8,9].

Gene co-expression modules tend to consist of genes that belong to the same cellular pathways or programs and help explain the global properties of the transcriptome as it relates to disease risk [10]. Networks-based co-expression module approaches have been used to identify causal variants in Late-Onset Alzheimer's disease [7,11]. However, such studies have failed to account for the heterogeneity of mechanisms that lead to complex diseases. Here, we analyze whole genome sequencing (WGS) and whole transcriptome data from three independent human cohorts from the Accelerating Medicines Partnership—Alzheimer's Disease (AMP-AD) Consortium. We use gene co-expression modules to develop quantitative phenotypes that account for the complex genetic architecture and heterogeneity of LOAD to more effectively map associated variants using genome-wide association. Furthermore, the method presented in this paper can be used to identify variants in other complex diseases.

## Results

### Description of post-mortem transcriptome study populations

**Fig 1:**
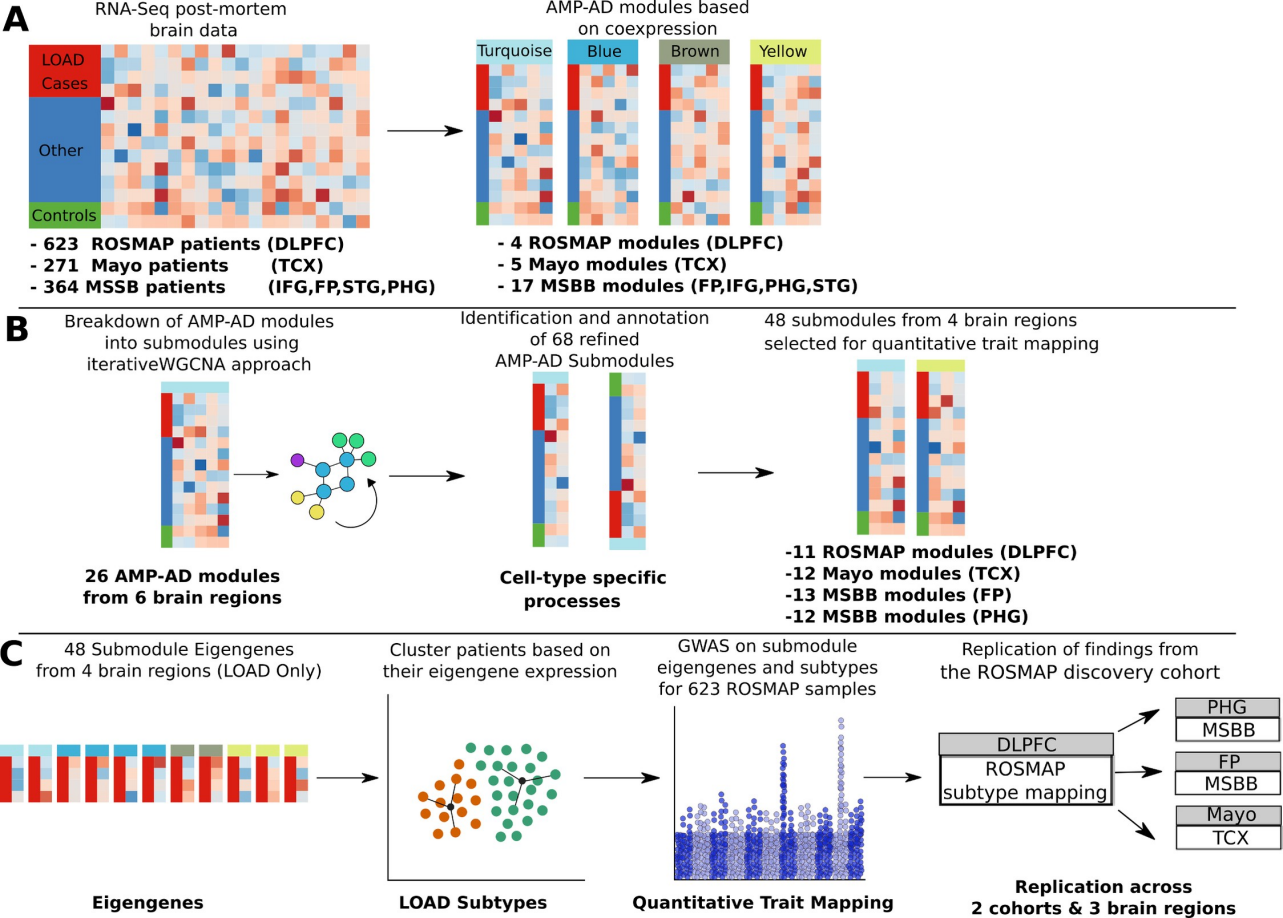
Method used in this study to map genetic drivers of LOAD pathology in the ROSMAP cohort. (A) RNA-Seq was performed on post-mortem brain samples from patients with Late-Onset Alzheimer’s Disease (LOAD). A modified procedure using seven different WGCNA protocols, followed by merging by clustering methods, was performed to obtain four modules based on gene co-expression. (B) Each of the 26 modules was subjected to iterativeWGCNA, a procedure that repeatedly performs WGCNA on expression data to generate highly correlated gene sets and exclude weakly correlated genes. 68 submodules were generated from the 26 modules. (C) The eigengene, or first principal component, was calculated for a subset of 48 submodules from four brain regions (DLPFC, TCX, FP, PHG) and used as a quantitative trait for single-variant association mapping. Furthermore, the eigengene expression for LOAD cases was used to perform cluster analysis and generate subtypes of LOAD cases. The Euclidean distance of each patient from each subtype centroid was used as additional quantitative trait–the subtype specificity metric–in a single-variant association mapping. ROSMAP mapping was used as the baseline, with the Mayo and MSBB cohorts serving as replicates.

To define novel quantitative phenotypes for LOAD, we obtained 26 post-mortem brain co-expression modules (doi.org/10.7303/syn11932957.1) that were harmonized from three independent cohorts of the AMP-AD consortium (Fig 1A, S1 Fig). This included post-mortem brain samples of 623 decedents from the ROSMAP cohort for the dorsolateral prefrontal cortex (DLPFC) brain region, 271 decedents from the Mayo cohort for the temporal cortex (TCX) brain region, and 364 decedents from the MSBB cohort for the frontopolar prefrontal cortex (FP), inferior temporal gyrus (IFG), parahippocampal gyrus (PHG) and superior temporal gyrus (STG) brain regions (Fig 1A). Approximately one-third of the patients were diagnosed with LOAD, while two-thirds were considered controls. The control group included elderly with normal cognition, as well as mild cognitive impairment and other forms of dementia. An overview of the post-mortem brain samples used in our analysis pipeline is provided in S1 and S2 Tables. Details on post-mortem brain sample collection, tissue and RNA preparation, sequencing, and sample quality control can be found in published work related to each cohort [12–14]. Multiple variables, such as sex, age of death, and sequencing batch effects were used as covariates in the normalization process to remove possible confounding factors across cohorts (Methods) [15].

### Refinement of 26 human co-expression modules identifies disease-associated transcriptomic signals

**Fig 2:**
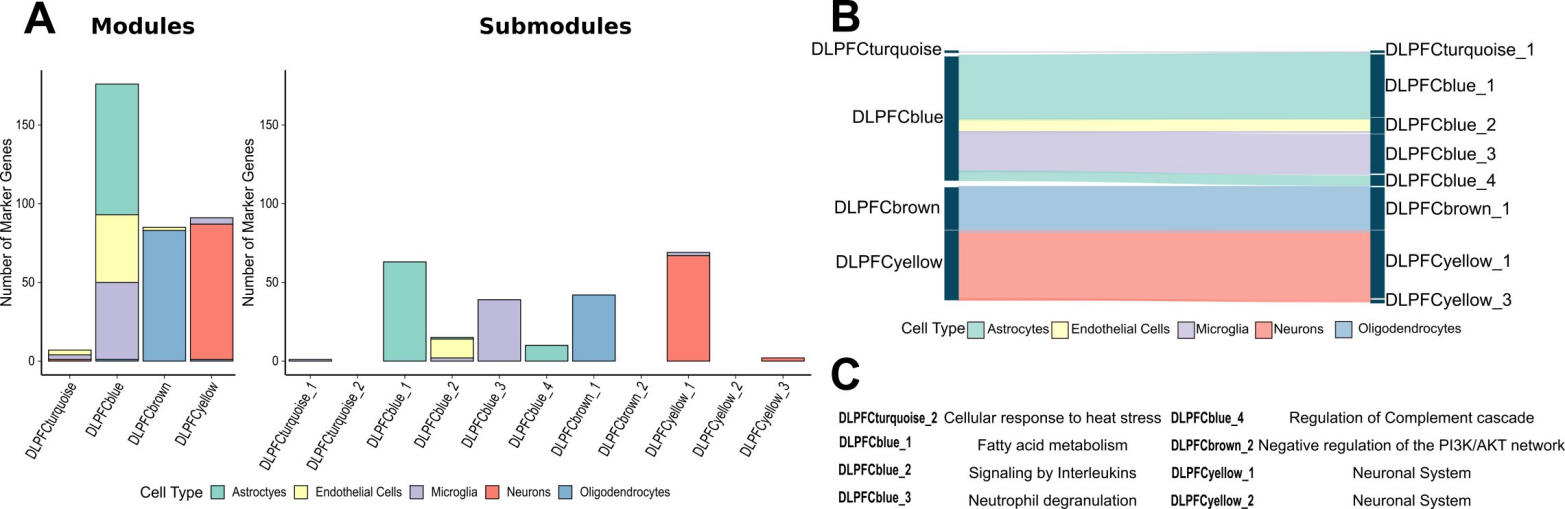
Cell-type specificity of modules is refined in submodules. (A) Cell-type specific marker genes reported by McKenzie *et al*. were used to annotate modules and submodules for astrocytes, endothelial cells, microglia, neurons, and oligodendrocytes. The top 100 marker genes for each cell-type were used. The iterativeWGCNA procedure generated submodules that were more cell-type specific than their modules of origin. (B) A Sankey diagram demonstrating which cell-type specific markers from modules were found in submodules for the ROSMAP cohort. (C) The top enriched Reactome pathways for ROSMAP submodules based on gene set enrichment analysis.

We performed an iterative gene list pruning process using the iterativeWGCNA approach [16] to refine the 26 human co-expression modules from the AMP-AD consortium (S3 Table). Each of these 26 modules contains several thousand co-expressed genes, implicated in multiple disease processes across multiple cell types. Therefore, it is often difficult to assign a cell-type-specific role for expression modules linked to a certain brain region. Our approach resulted in 68 distinct subsets, or submodules, of highly correlated genes that were exclusive to each module (Fig 1B, S4 Table). Genes that were not highly correlated to any submodule were removed since they are less likely to contribute to the overall signal of the submodule and more likely to introduce noise. We then annotated the 68 co-expression submodules to identify molecular pathways and processes that are significantly enriched within submodules across the six brain regions from the three independent LOAD cohorts (S2 Fig). Pathway enrichment analysis was performed using GO terms, KEGG pathways, and Reactome pathway data sets to highlight the biological specificity of co-expression signals captured by the different submodules (S5 Table). We identified multiple functional consensus clusters across the 68 submodules, which showed a significant overlap in functional enrichment for similar biological pathways and processes across the six brain regions (S2 Fig). These functional consensus clusters associated with the 68 submodules revealed gene sets for specific biological pathways, including tau-protein kinase activity, neuroinflammation, myelination, and cytoskeletal reorganization (S2 Fig). Furthermore, incorporating information from previously defined cell-type-specific markers derived from bulk and single cell RNA Sequencing (RNA-Seq) [17] showed that refining the 26 co-expression modules into 68 submodules resulted in multiple novel submodules enriched for cell-type-specific markers (Fig 2, S3 Fig).

### Single-variant association mapping of submodule eigengenes across cohorts

To map the genetic drivers of biological disease-associated signals resolved by submodules, we performed single-variant association mapping of submodule eigengenes (Fig 1C) using whole-genome sequencing data from the AMP-AD knowledge portal (doi.org/10.7303/syn10901601). We then applied a variance component linear mixed model implemented in the EMMAX software to identify novel genetic loci associated with submodule eigengenes (Methods) [18]. Eigengenes were defined as the first principle component of the gene expression data associated with each of the 68 submodules. They capture most of the variation in gene co-expression and reduce noise associated with the transcriptomic data. We included eigengene expression data from four of the brain regions (TCX, PHG, FP, DLPFC), focusing on tissues from the frontal cortex, temporal cortex, and hippocampus due to their relevance to LOAD neuropathology [19]. QQ plots indicate minimal effects of genomic inflation, and consequently population substructure, on the analyses (S4 Fig).

**Fig 3:**
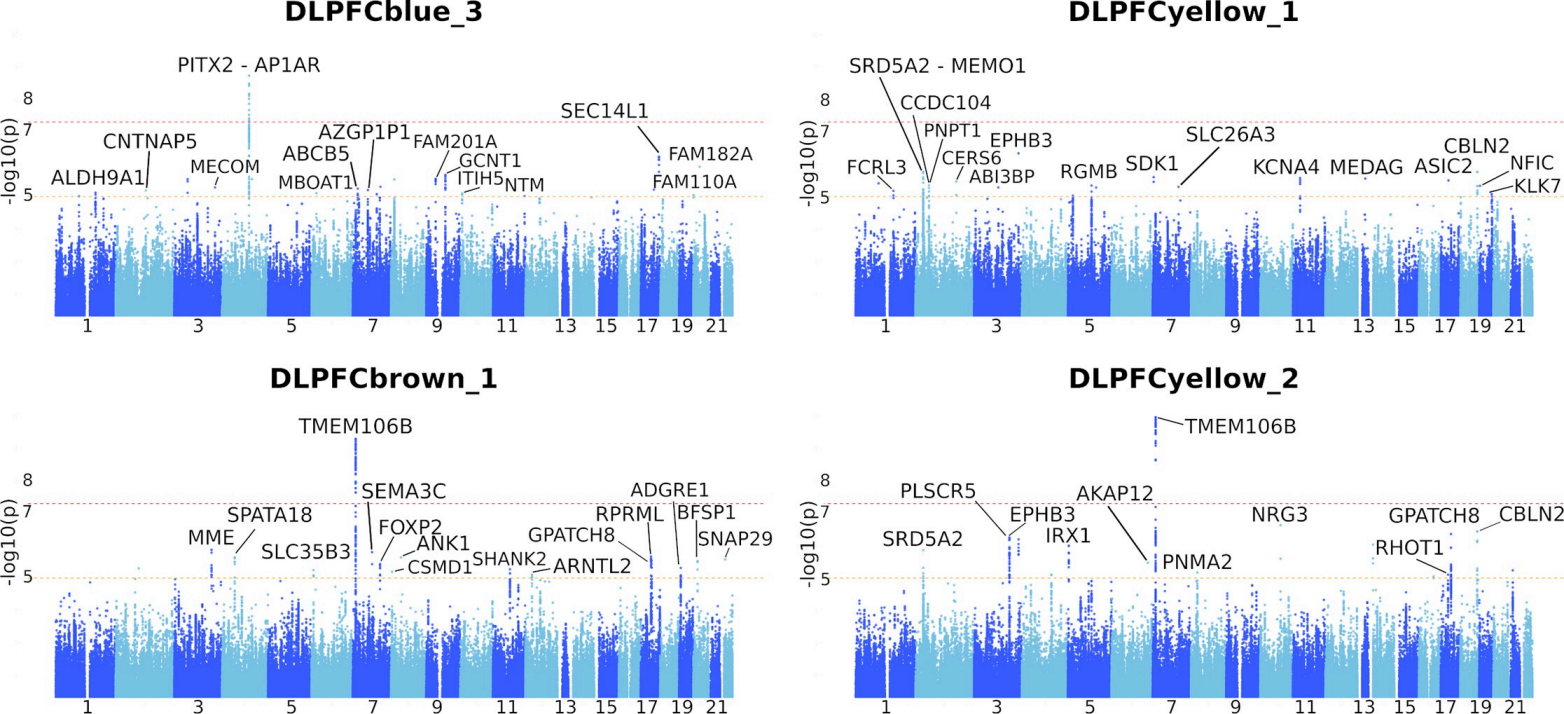
Manhattan plots of single-variant association of select submodule eigengenes in ROSMAP. Eigengene expression for each submodule was used as a quantitative trait when performing single-variant mapping. Multiple submodule eigengenes were associated with SNPs at a genome-wide significance level of p = 5×10^−8^ (red dotted line). Loci of interest are annotated with the gene closest to the region. Some SNPs were also detected at a genome-wide suggestive level of p = 1×10^−5^ (yellow dotted line). DLPFCblue_3 contains genes related to the TREM2/TYROBP pathway, an important network of genes related to microglial activation during neuroinflammation of the brain. Submodules were associated with both unique and overlapping loci. For example, DLPFCbrown_1 and DLPFCyellow_2 are derived from separate co-expression modules but were both associated with the *TMEM106B* locus. Similarly, DLPFCyellow_1 and DLPFCyellow_2 were derived from the same co-expression module but were associated with a mix of overlapping and unique loci.

Genome-wide suggestive and significant loci were detected for submodules in the four brain regions (Fig 3, S6–S9 Tables). We identified multiple loci that were replicated across the three cohorts at a genome-wide significant level. For instance, rs1990620 is a known LOAD-associated variant in *TMEM106B* that was identified as genome-wide significant in the DLPFC region from the ROSMAP cohort and was replicated (p < 5×10^−2^) in the remaining three brain regions from the Mayo and MSBB cohorts. This highlights the usefulness of our newly derived quantitative phenotypes to identify genetic variants associated with specific co-expression submodules driving disease pathology.

### Stratification of LOAD cases based on 68 AMP-AD co-expression submodules

**Fig 4:**
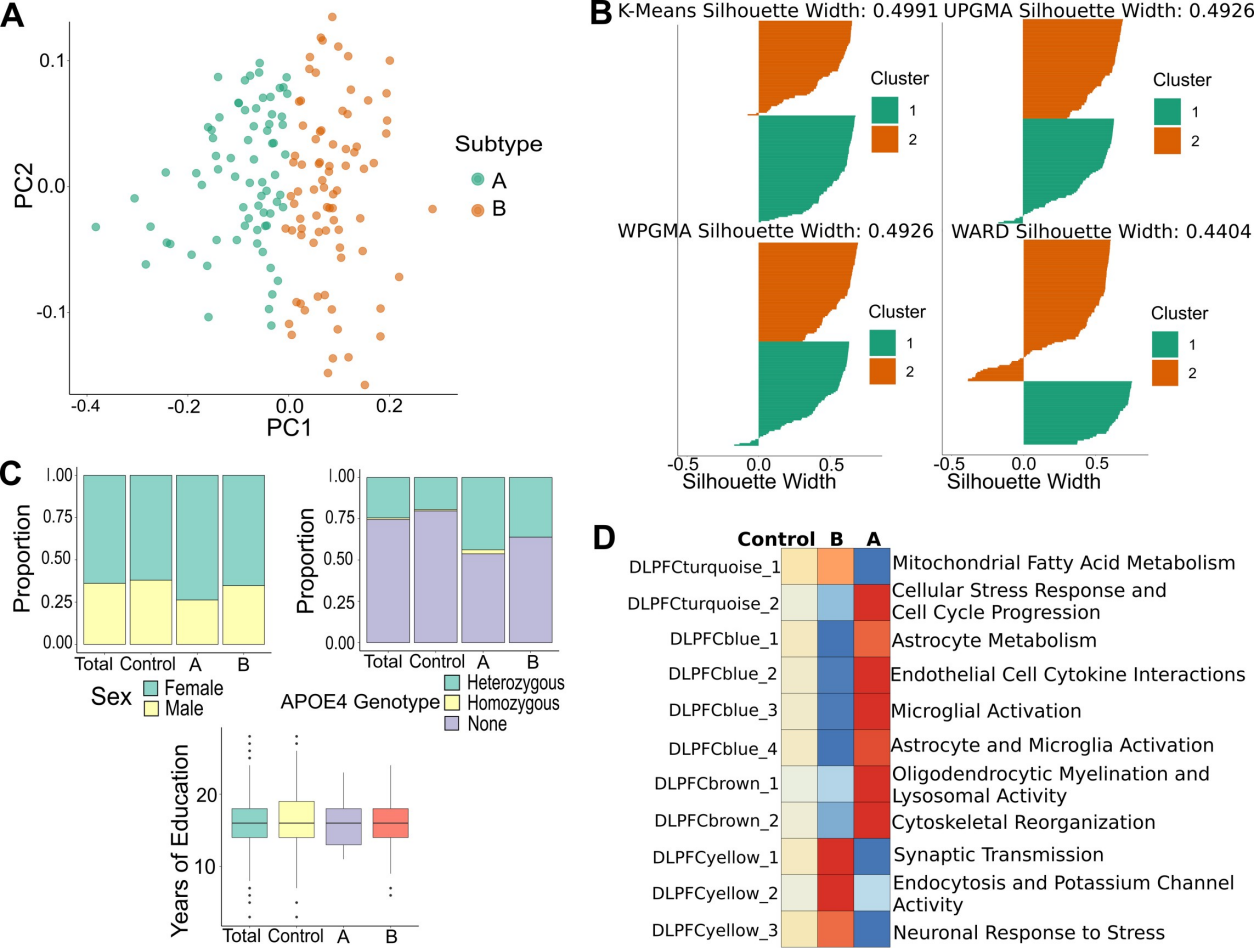
Clustering on eigengene expression in ROSMAP data generates two subtypes. (A) Eigengene expression was used to cluster LOAD cases into subtypes using K-Means clustering for the DLPFC region. The number of clusters were determined by democratizing results across 30 mathematical indices using the NbClust R package. Two clusters with similar number of cases were generated. (B) Silhouette plots were generated by four different clustering methods. The mean silhouette width of a cluster represents how similar objects are to the centroid of the cluster, and the mean silhouette width of all objects represent how well the data have been clustered. In the case of ROSMAP, the K-means method had the highest mean silhouette width across all LOAD cases. We performed a similar analysis for other brain regions. (C) No significant differences in proportion of sex, *APOEε4* genotype, and years of education between subtypes. (D) A strong immune and neuronal signal in the scaled eigengene expression profile of the subtypes compared to control decedents (including MCI).

We next assessed if submodule composite phenotypes could be used to better account for the observed heterogeneity in the genetic architecture of LOAD by clustering patients based on their co-expression profiles (Fig 1C). Clustering was performed to determine subtypes of LOAD cases for four brain regions (TCX, PHG, FP, DLPFC). The NbClust R package was used to identify the optimal number of clusters for different clustering methods by polling with the majority rule across 30 indices [20]. The NbClust package identified two subtype clusters for the ROSMAP (DLPFC region) and MSBB cohorts (FP, PHG regions), while three clusters were observed for the Mayo cohort (TCX region). An example for the ROSMAP cohort is shown in Fig 4. The number of cases in each identified subtype cluster was balanced across all three cohorts (S10 Table). A comparison of the different methods in terms of cluster assignment indicates that results are not affected substantially by the choice of clustering method in either of the cohorts (S11 Table). Notably, our newly defined molecular subtypes were not enriched for common LOAD-associated covariates, such as sex, *APOEε4* genotype, or years of education (Fig 4C, S5 Fig). Furthermore, eigengene expression profiles for each subtype were used to assess the association of each subtype with molecular and biological pathways associated with submodules (Fig 4D). We observed no significant enrichment of cognitive or neuropathological measures between the subtypes for the DLPFC region (S5 Fig).

### Single-variant association mapping of subtype specificity metric in ROSMAP

In order to determine genetic variants associated with subtype classification, we used the Euclidean distances of each patient from the centroid of each subtype as a quantitative trait for genetic mapping. We performed genome-wide mapping for LOAD subtype association using the 623 patients of the ROSMAP cohort due to the larger sample size when compared to the MSBB and Mayo cohorts.

**Fig 5:**
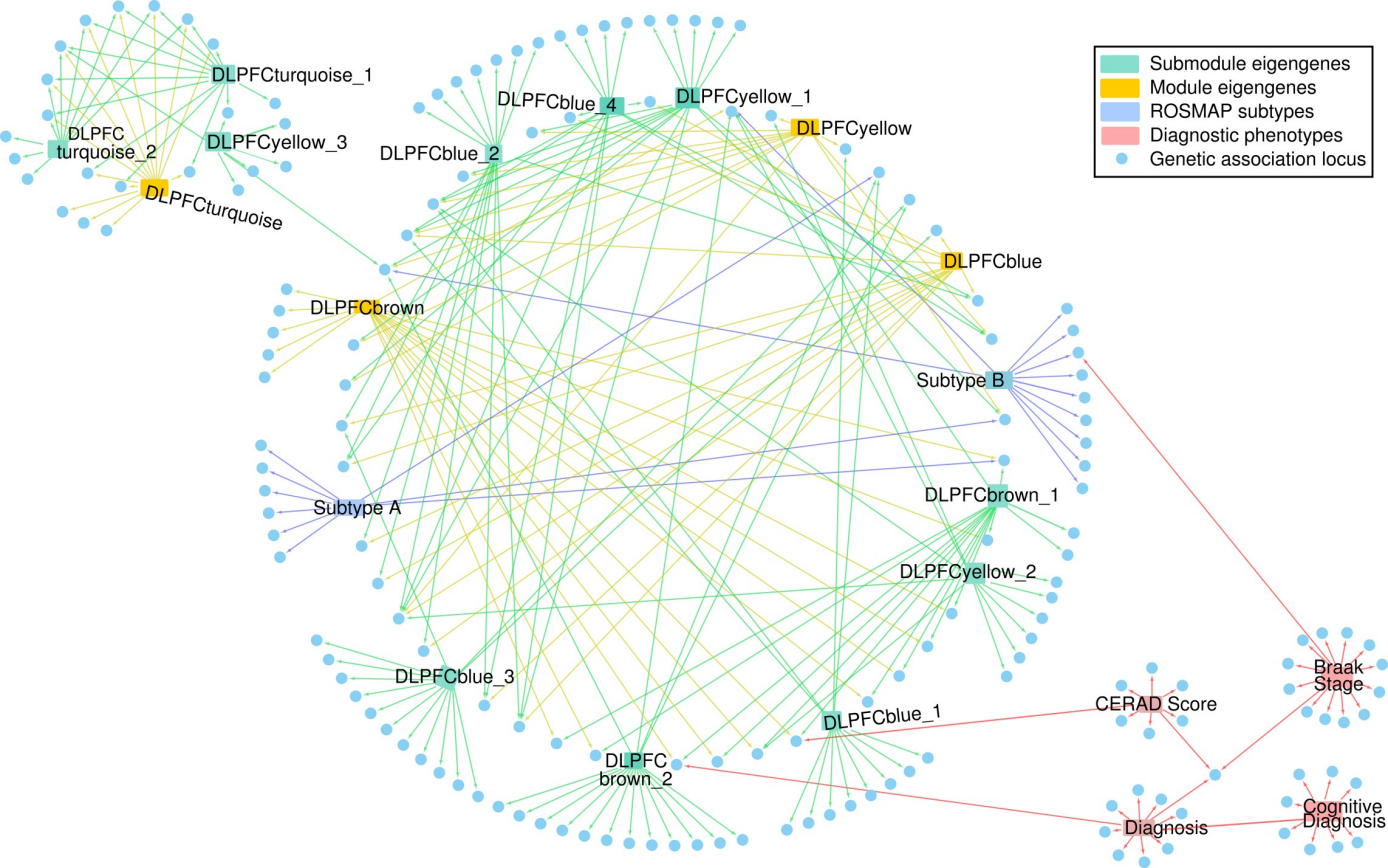
Network of endophenotypes and associated loci. We created a directed network describing the loci detected from the multiple analyses in this study. Blue nodes represent loci. Red nodes represent phenotypes. An edge from a phenotype to a genetic locus signifies that the locus is associated with that phenotype. Diagnostic phenotypes (red edges; right) were associated with some of the loci detected in this study. The module eigengenes (yellow edges), submodule eigengenes (green edges), and subtypes (blue edges) were associated with both overlapping and unique loci (center and left). A community of loci was associated with multiple submodules associated with microglia, endothelial cells, astrocytes, and oligodendrocytes (center). A small community of loci was associated with submodules related to proteostasis (left).

Genome wide association mapping revealed various significant variants across subtypes in ROSMAP decedents (S6 Fig, S9 Table). Several variants in *TMEM106B* reached our genome-wide significance threshold after multiple testing correction (p < 5×10^−8^). *TMEM106B* is a known modifier of neurodegenerative disease and cognitive aging, which has been previously linked with cognitive performance [21]. Among the variants we identified in *TMEM106B*, one genome-wide suggestive allele was identified for LOAD Subtype B (p < 4×10^−6^, rs1990620^G^) in ROSMAP. This association with the protective rs1990620^G^ variant reached a genome-wide significant level with three of our previously mapped co-expression submodules from the ROSMAP cohort (Fig 3): DLPFCbrown_2 (p = 3.72x10^-07^), DLPFCbrown_1 (p = 8.91x10^-11^), and DLPFCyellow_2 (p = 5.88x10^-14^). The DLPFCbrown_1 submodule is enriched for genes related to myelination and lysosomal activity (KEGG pathways hsa00600 and hsa04142), while DLPFCyellow_2 is enriched for genes related to endocytosis and potassium channel activity (KEGG pathway hsa04144 and Reactome pathway R-HSA-1296071). We replicated the subtype specific association of the protective rs1990620^G^ variant in the TCX brain region from Mayo cohort (Subtype B, p = 0.041), while we did not observe a significant association with variants in *TMEM106B* in the FP and PHG brain regions in the MSBB cohort. To our knowledge, this is the first report associating protective *TMEM106B* variants with molecular LOAD endophenotypes that link disruption of lysosomal and myelination pathways to disease subtypes. This is in line with results from a study in mice which showed that loss of *TMEM106B* function rescued lysosomal phenotypes related to frontotemporal dementia [22]. Furthermore, the identified protective allele rs1990620^G^ disrupts a known CCCTC-binding factor (CTCF) site, which has been shown to modify the inflammatory response in the course of aging [23]. Differential expression analysis of haplotype carriers of the protective rs1990620^G^ variant in *TMEM106B* showed an up-regulation of neuroactive ligand receptor interactions, while decedents carrying the risk variant showed significant up-regulation for pathways related to neuroinflammation (KEGG pathway hsa04380) (S7 Fig). Besides the association with *TMEM106B* in Subtype B, protective variants near *MTUS2* were identified which are in close vicinity to *HMGB1*, a locus that has been previously implicated in brain atrophy [24]. In order to provide a better overview of the associated loci, we generated a directed network to visualize loci that were associated with different modules, submodules, subtypes, and diagnostic criteria for the ROSMAP cohort (Methods). Interestingly, we observed that while certain loci were uniquely associated with single modules or submodules, a community of shared loci was associated with modules and submodules annotated for microglia, endothelial cells, astrocytes, and oligodendrocytes (Fig 5). A separate community of loci was associated with modules and submodules annotated for proteostasis (Fig 5). Many of the loci associated with diagnostic criterion in the ROSMAP cohort were independent from these two communities (Fig 5). Only one locus was identified which showed a suggestive association with both Braak stage and Subtype B in our analysis.

### Suggestive SNPs in ROSMAP are replicated in the Mayo and MSBB cohorts

To assess the validity of our genetic findings in ROSMAP, we aimed to replicate our results across three brain regions from the Mayo (TCX brain region) and MSBB (PHG, FP brain regions) cohorts (Fig 1C). In addition, we compared our results to a catalog of recently published GWAS results in order to evaluate the novelty of our findings.

A total of 1326 unique variants representing 163 loci reached a genome-wide suggestive or significant p-value (p < 1×10^−5^) in the DLPFC region when pooled from all 11 DLPFC eigengenes and two subtype-specific variant mapping analyses (S12 Table). Of these, 645 SNPs were replicated in the PHG analyses, 762 SNPs were replicated in the FP analysis, and 482 SNPs were replicated in the TCX analyses (replication threshold, p < 1×10^−2^). Overlapping co-expression submodules across brain regions (S2 Fig) were associated with similar loci.

Of the 1326 variants identified in ROSMAP, 29 variants have also been previously reported in the NHGRI-EBI catalog (S13 Table). In each case, the most significant SNP from a prior study was a suggestive SNP in the DLPFC region. Fifteen of these 29 previously reported variants were suggestive SNPs at the *TMEM106B* locus in the DLPFC region. These 15 variants were previously reported for association with traits such as depression [25–27], neuroticism [26,28–31], coronary artery disease [32], and frontotemporal dementia [33]. The *TMEM106B* variant associated with dementia, rs1990620, was replicated with submodule eigengene expression in three out of four brain regions (DLPFC, TCX, PHG) in the AMP-AD cohorts (S12 and S13 Tables). An *ITGA2B* variant (rs5910), previously associated with Parkinson’s disease [34], was replicated in the TCX and PHG regions (S12 and S13 Tables). Three suggestive ROSMAP variants at the *LMX1B* locus were previously reported for association with glaucoma [35–37], and replicated in the TCX and FP regions (S12 and S13 Tables). Taken together, however, a significant number of the 163 loci detected in the ROSMAP cohort implicated novel variants in LOAD processes, many of which were replicated in brain regions from the Mayo and MSBB cohorts.

### Molecular LOAD subtypes differ in their inflammatory response

**Fig 6:**
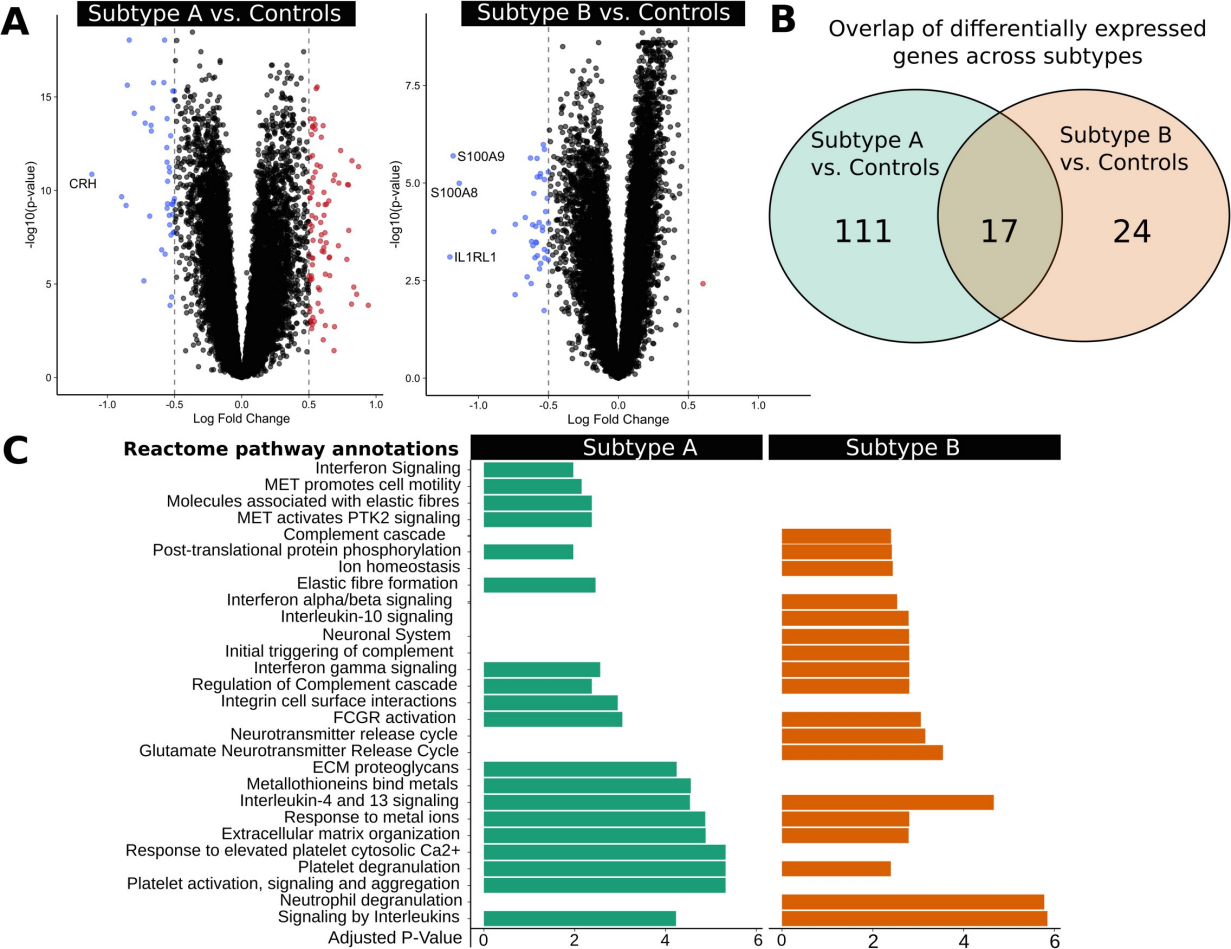
Differential expression analysis of ROSMAP subtypes reveals heterogeneity in inflammatory response in LOAD cases. (A) Differential expression analysis comparing each subtype to control decedents for the DLPFC region was performed using the limma R package. We show up-regulated (red, p < 0.05, log fold change > 0.5) and down-regulated (blue, p < 0.05, log fold change < -0.5) genes in the volcano plot and label genes that have an absolute log fold change > 1 (dotted lines). (B) Differentially expressed genes (p < 0.05, absolute log fold change > 0.5) show a partial overlap between subtypes. (C) Top Reactome pathways for differentially expressed genes for both subtypes are reported. Subtype A demonstrates an enrichment of immune and stress-response related pathways across up-regulated genes, while Subtype B demonstrates a down-regulation of a set of specific immune-related pathways linked to S100A8/A9 activation.

To better understand the underlying molecular differences across the novel LOAD subtypes, we performed differential expression analysis for each subtype against a set of controls in the ROSMAP cohort (Fig 6A, S8 Fig). The set of controls included 471 decedents who were either cognitively normal or had mild cognitive impairment. Performing this analysis without the mild cognitive impairment cases yielded essentially the same results (S9 Fig). The Venn diagram in Fig 6B depicts the comparison across the two subtypes. Interestingly, we found that cases associated with Subtype A showed a stronger transcriptional response with 127 differentially expressed genes (adjusted p < 0.05, absolute log fold change > 0.5) when compared with controls. Among the most significantly down-regulated genes associated with Subtype A cases was the stress-response mediator corticotropin-releasing hormone (*CRH*; Fig 6A). Overacting *CRH* signaling has been implicated in inflammatory disorders and LOAD where it has been proposed as a therapeutic target to reduce the negative effects of chronic stress related to memory function and amyloid beta (Aβ) production [38]. Cases associated with Subtype B had 40 differentially expressed genes (FDR adjusted p < 0.05, absolute log fold change > 0.5), 39 of which were down-regulated when compared to controls. Notably, we found that two key pro-inflammatory mediators of amyloid deposition (*S100A8*, *S100A9*) were among the most significantly down-regulated genes in Subtype B decedents when compared to controls (Fig 6A). Both genes, which are established inflammatory biomarkers, are part of a complex that serves as a critical link between the amyloid cascade and inflammatory events in LOAD [39]. Furthermore, we identified multiple pathways linked to *S100A8/9* activation, including IL-10 signaling and complement activation, that were enriched across down-regulated genes in Subtype B but not in Subtype A (Fig 6C). In addition, molecular pathways linked to microglia activation, the immune response, and the stress response were found among the most significant pathways and gene sets that differ across subtypes (S8 Fig, S14 Table). This highlights that our LOAD subtypes differ in their inflammatory response and that known LOAD biomarkers might be used to stratify patients based upon their inflammatory response to the observed disease state. The same analysis in the Mayo and MSBB cohorts revealed that the corresponding subtypes can also be distinguished based on their inflammatory response (S10 Fig). However, the signal derived from the molecular pathway expression profiles in both the Mayo and MSBB cohorts is not as strong as in the ROSMAP cohort, which is likely due to the smaller sample size and differences in population structure across both cohorts. Although inflammatory markers were the most differentially expressed, the subtypes are characterized by diametric associations with the eigengenes of multiple submodules annotated for different pathological processes (Fig 4D). Thus, the detection of differentially expressed inflammatory markers between subtypes is likely because inflammation is the strongest post-mortem signal present in transcriptomic data.

## Discussion

Common, complex diseases such as LOAD are characterized by phenotypic heterogeneity and the presence of multiple common variants affecting disease risk. In this study, we present an analysis that uses transcriptomic co-expression data and whole-genome sequencing from multiple cohorts to dissect phenotypic heterogeneity and identify potential genetic drivers of complex trait pathology in LOAD.

Here, we used an iterative pruning approach based on 26 human post-mortem co-expression modules to generate 68 novel submodules that contained genes associated with LOAD specific biological pathways and molecular processes. Indeed, we observed that genes in the novel submodules are enriched for functional terms that were specific to pathways associated with LOAD, such as lipid modification, the TREM2/TYROBP signaling axis, and tau-protein kinase activity. Furthermore, submodules from distinct brain regions clustered independently, suggesting that the genes captured in each submodule represented signals that were associated with LOAD pathology rather than cohort- or tissue-specific factors. Notably, our pruning approach identified submodules which were much more specific for markers of different brain cell types when compared to the initial co-expression modules. This is in line with recent studies showing that different cell types in the brain play specific roles at different stages in the pathogenesis of LOAD [40]. Taken together, our results demonstrate that the novel human co-expression submodules identified in this study capture cell-type-specific pathways associated with LOAD pathogenesis in the brain.

Mapping the eigengene expression for individual submodules represents a pathway- or process-level alternative to expression quantitative trait locus (eQTL) mapping for each individual transcript. Since the human co-expression submodules represented pathological, cell-type-specific pathways in LOAD brain tissue, mapping eigengene expression for decedents was expected to identify genetic drivers of LOAD pathology. RNA-Seq data from post-mortem brain tissue in human cohorts contains a strong immune signal, as evidenced by repeated identification of genetic loci related to microglial response in meta-analyses with increasingly large cohorts [5,41]. Using submodule eigengenes as quantitative traits for single-variant association provided an opportunity to identify genetic drivers of biological processes that are known to be drivers of early LOAD pathogenesis, such as astrogliosis, neuronal plasticity, myelination, and vascular blood brain barrier interactions [40]. Suggestive variants identified were unique to subsets of submodules. For instance, the *TMEM106B* locus was associated at a genome-wide significant level with the DLPFCbrown_1 and DLPFCyellow_2 eigengenes (Fig 3), representing processes related to oligodendrocytic myelination, lysosomal activity, endocytosis, and potassium channel activity. This novel association between protective variants in *TMEM106B* with molecular LOAD endophenotypes linked to lysosomal and myelination dysfunction is potentially of great interest. The *TMEM106B* locus has been implicated in cognitive aging, with functional consequences in frontotemporal dementia related to lysosomal activity [21–23]. A recent transcriptome study implicated protective *TMEM106B* variants in differences in neuronal proportions across LOAD patients, supporting the idea that impaired lysosomal function may lead to a toxic buildup of waste in the cell, a common process among many neurodegenerative disorders [42]. Therefore, the presence of *TMEM106B* variants in combination with other risk factors might alter the course and severity of neurodegeneration across patient subtypes. Furthermore, we identified multiple loci associated with a module linked to microglia function (DLPFCblue_3). This co-expression module contains members of the TREM2/TYROBP signaling pathway, an important mediator of neuroinflammation. Variants in the *FAM110A (*rs1014897*)*, the *CNTNAP5 (*rs76854344*)* and *NTM* (rs1040103*)* genes associated with this inflammatory module have been previously linked to posterior cortical atrophy, LOAD [43], and white blood cell count [44]. Taken together, we show that quantitative trait mapping using submodule eigengene expression can identify novel genetic variants impacting relevant disease pathways.

Eigengenes represent a dimensional reduction of transcriptomic data onto axes of pathological relevance. Thus, we expected that clustering on the eigengene expression of LOAD cases would generate pathway-level profiles of putative molecular LOAD subtypes based on case heterogeneity. We observed that average eigengene expression of different subtypes was enriched for different submodules in the four brain regions for which subtype analysis was performed, an example of which is presented for the DLPFC region in Fig 4. Similar diametric enrichment patterns were identified in the remaining brain regions (S10 Fig). These results suggest that the biological programs identified by submodules in this study align themselves along the heterogeneity of transcriptomic data present in LOAD cases across multiple cohorts rather than differentiating solely based on cases and controls. Furthermore, the stratification of patients based on submodule expression profiles demonstrated that there is significant variation in immune response in post-mortem brain tissue (Fig 6, S7 Fig), a process that is considered a hallmark of LOAD pathogenesis. Variants associated with the subtype specificity metric overlapped with the variants associated with individual submodule eigengenes (Fig 5). This suggests that the genetic factors influencing subtypes can be dissected into loci driving specific submodules. Such dissection of genetic loci can provide the basis for more targeted treatment of dysfunctional pathways that contribute to different subtypes of LOAD.

Our subtypes in the DLPFC brain region of the ROSMAP cohort represent differences in transcriptomic profiles of LOAD cases derived from post-mortem RNA-Seq data. A lack of temporal data makes it challenging to decisively interpret these profiles derived from post-mortem brain samples. The identified subtypes may represent distinct LOAD endpoints, differences in disease severity, environmental effects, or phases of molecular pathology. Neither of our novel subtypes was associated with cognitive or neuropathological outcome (S5 Fig). Furthermore, covariates such as sex, *APOE* genotype, and years of education were not significantly enriched in any given subtype (Fig 4C). This suggests that the transcriptomic profiles do not represent transitions in disease severity and that there are overall risk factors not reflected in transcriptomic subtypes. Furthermore, both subtypes are associated with unique loci that belong to the same community of loci detected by submodule mapping (Fig 5), indicating that the subtypes capture various combinations of genetic elements that lead to LOAD pathology. While suggestive, these transcriptomic LOAD subtypes will require further validation in cohorts that adequately account for disease progression.

The methodology presented in this study is not limited to RNA-Seq data and can be performed on other omics datasets, such as proteomics or metabolomics. As such data become available for the decedents in these cohorts, this analysis can be expanded across these additional informative dimensions.

## Methods

### Whole genome sequencing and RNA sequencing data

We obtained whole-genome sequencing and RNA-Seq data from Synapse (https://www.synapse.org/) for three cohorts from the AMP-AD consortium: the Mayo Clinic, Mount Sinai Brain Bank, and Rush University. The Mayo Clinic (Mayo) cohort consists of 276 temporal cortex (TCX) samples from 312 North American Caucasian subjects consisting of cases characterized with LOAD, pathological aging (PA), progressive supranuclear palsy (PSP), or elderly controls [13] (doi.org/10.7303/syn5550404). The Mount Sinai Brain Bank (MSBB) cohort consists of 214 frontopolar prefrontal cortex (FP), 187 inferior temporal gyrus (IFG), 160 parahippocampal gyrus (PHG), and 187 superior temporal gyrus (STG) samples characterized with LOAD, elderly control, or mild cognitive impairment (MCI) (doi.org/10.7303/syn3159438). The Rush University's Religious Orders Study and Memory and Aging Project (ROSMAP) cohort consists of 623 dorsolateral prefrontal cortex (DLPFC) samples of individuals from 40 groups of religious orders from across the United States (ROS) and older adults in retirement communities in the Chicago area (MAP), characterized with LOAD, elderly control, or MCI [7,45] (doi.org/10.7303/syn3219045). A summary of samples from each of the cohorts is provided in S1 and S2 Tables. Sex, age of death, and batch were used as covariates for normalization in the ROSMAP and Mayo data. Sex, age of death, race, and batch were used as covariates for normalization in the MSBB data.

### Co-expression modules and iterativeWGCNA

Data on human AMP-AD co-expression modules were obtained for the three cohorts from Synapse (doi.org/10.7303/syn11932957.1). A recent study has identified these modules as part of a transcriptome wide LOAD meta-analysis [15]. In brief, a modified procedure using five different co-expression analysis protocols followed by graph clustering methods was performed to obtain 30 modules across all three cohorts (doi.org/10.7303/syn2580853), 26 of which corresponded to the six tissue regions used in this study. A summary of these modules is provided in S3 Table. We focused on tissues from the frontal cortex, temporal cortex, and hippocampus due to their relevance to LOAD neuropathology [19]. These modules are generally large, containing thousands of genes that represent multiple functions [15]. In order to construct more functionally-specific submodules from these AMP-AD co-expression modules, we subjected them to a repeated pruning process called iterativeWGCNA [16], which includes performing WGCNA on each AMP-AD co-expression module independently. The gene sets produced by this process were then pruned to ensure that only highly correlated genes remained by evaluating the connectivity of the genes to the gene set eigengene. The resulting gene sets, containing highly correlated genes, were combined and the process was repeated until the gene sets converged. The algorithm then attempted to reclassify genes from the residual gene set into submodules. We specified a soft-threshold power of six, a minimum eigengene connectivity of 0.6, and a required module size of 100 to promote the generation of submodules that capture pathway-level signals. The final set of 68 submodules consisted of highly correlated and cell-type-specific genes. The submodules were mutually exclusive for a given cohort but overlapped with submodules from other cohorts. A summary of these submodules is provided in S4 Table. An eigengene for a given submodule is defined as the first principle component of gene expression data within each submodule.

### Stratification of LOAD cases based on clustering of human co-expression submodules

Eigengene expression data for TCX, PHG, FP, and DLPFC regions was used to stratify LOAD cases into subtypes based on each brain region separately. We used the NbClust R package to determine the optimal number of clusters across different clustering methods by polling with the majority rule across 30 indices [20]. We tested agglomerative hierarchical approaches (Ward, UPGMA, WPGMA) and a reallocation approach (K-means) on the eigengene expression data and evaluated the within-cluster similarity of cases using silhouettes. The silhouette score of a given object (data point) is a measure that simultaneously assesses how similar this object is to its cluster and how different it is from all the other clusters [46]. A simulation study suggests that no one clustering method outperforms the other consistently and that mean silhouette widths can be used to pick the ideal clustering method and compare clustering across datasets [47]. The silhouette plots revealed that different methods were required for the different regions to generate clusters with the largest average silhouette widths. We determined that K-means was an optimal approach for DLPFC, Ward was optimal for PHG and TCX, and UPGMA was optimal for FP after analyzing silhouette plots of clusters generated by each method for each region. An example of silhouettes used to determine the ideal clustering method for the DLPFC region is shown in Fig 4. A summary of the clusters for each brain region, considered case subtypes, is provided in S10 Table. In the subtypes generated for the DLPFC region from the ROSMAP cohort, we assessed each subtype for enrichment of cognitive and pathological measures. We used Braak stages as a measure of neurofibrillary tangle burden and CERAD scores as a measure of neuritic plaque burden [48,49]. We also assessed the rate of decline in memory, executive function, visuospatial function, and language across the subtypes. Definitions, collection, and standardization of these decline measures can be found in previously published work [50].

### Differential expression analysis of case subtypes

For differential expression analysis, control decedents were defined as cognitively normal and MCI decedents for PHG, FP, and DLPFC. In the case of TCX, control decedents included cognitively normal, PSP, and PA decedents. For each of the regions used to stratify LOAD cases (TCX, PHG, FP, and DLPFC), we performed differential expression analysis to compare gene expression in LOAD subtypes with control decedents as described above. We repeated this analysis excluding MCI, PSP, and PA decedents from the control group and got essentially the same results (S9 Fig). We used the limma R package to perform the differential expression analysis between subtype and control decedents [51]. We used the clusterProfiler R package to perform KEGG and Reactome pathway analysis on differentially expressed genes to determine the signal captured by clustering on eigengene expression data [52].

### Single-variant association of eigengene expression and subtype specificity

We used EMMAX [18], a variance component linear mixed model, to perform single-variant association, using each submodule eigengene as a quantitative trait. In addition, we developed a subtype specificity metric for each brain region by calculating the Euclidean distance between the eigengene expression profile of each decedent and the centroid of each subtype cluster. This resulted in a vector of scores for each subtype that was mapped as a separate trait. All quantitative trait mapping results had a genomic inflation factor near one, indicating that there was no significant population substructure effect on the mapping. QQ plot analysis on the p-values showed no evidence of population substructure or confounding effects (S4 Fig).

### Replication of suggestive and significant SNPs in other cohorts

The ROSMAP cohort represented the most adequately powered cohort in the study and was therefore used as our baseline, while the other cohorts were utilized for assessing replication of suggestive and significant SNPs. SNPs were considered suggestive with a p-value smaller than 1×10^−5^ and genome-wide significant with a p-value smaller than 5×10^−8^, which are standard cutoffs for GWAS. Suggestive and significant SNPs from the DLPFC region in ROSMAP were considered replicated in the TCX, FP, and PHG regions if the SNPs were associated with the submodule eigengenes or subtype specificity metric of the given region at a p-value of 0.05. In addition, we compared the ROSMAP loci to prior association studies using summary statistics obtained from the NHGRI-EBI catalog [53]. Loci were considered replicated in this case if suggestive and significant SNPs from the ROSMAP cohort were reported in these studies at a p-value smaller than 5×10^−8^ (Fig 1C and phase 3 in S1 Fig).

### Network of loci and associated quantitative phenotypes in ROSMAP

We built a directed network of quantitative phenotypes and associated loci to better visualize the communities of loci that were associated with our newly derived quantitative phenotypes in the ROSMAP cohort (the subtype specificity metric and submodule eigengenes), including both suggestive and significant loci. We included in this network results of single-variant association of diagnostic criteria for other relevant traits, including module eigengenes, Braak stage, CERAD scores, cognitive diagnosis, and case-control diagnosis (using EMMAX [18] as for the other traits). The network was built in Cytoscape version 3.7 (https://cytoscape.org/) [54] and the nodes were organized using the “Circular Layout” option. The color of the edge was used to distinguish the type of association (red for diagnostic criteria, blue for subtype, yellow for module, and green for submodule).

## Supporting information

S1 Fig The complete analysis carried out in this study is divided into three phases. Phase 1 involved the co-expression analysis of ROSMAP and other cohorts to generate submodules representing biological processes involved in Alzheimer’s pathology. Eigengene expression from the submodules were used to perform single-variant association and identify loci that act as putative genetic drivers of these biological pathways. Phase 2 involved the clustering of LOAD cases in ROSMAP and other cohorts based on an agnostic clustering method. Subtypes were mapped using single-variant association to identify loci that may explain the heterogeneity observed in LOAD cases. Phase 3 involved the replication of genome-wide suggestive or genome-wide significant SNPs from the ROSMAP cohort in other tissue regions and previous studies. SNPs were replicated in three other tissue regions (PHG, FP, TCX) and in 27 studies from the NHGRI-EBI catalog.
(TIF)

S2 Fig Clusters of modules and submodules based on gene overlap reveal cell-type and functional signatures. (A) A previous study by Logsdon *et al*. reported 5 consensus clusters across 7 tissue regions based on the modules generated for each tissue region. A Jaccard matrix heatmap is used to visualize the overlap of genes in each module between tissue regions and cohorts. (B) Submodules were divided into 15 functional clusters based on hierarchical clustering that demonstrated specificity for certain biological pathways. These functional clusters formed independently of module of origin and tissue of origin.
(TIF)

S3 Fig Functional consensus clusters demonstrate cell-type specificity. Brain tissue cell-type specific markers reported previously by McKenzie *et al*. were used to assess the cell-type specificity of modules and submodules. Consensus clusters B broadly captured astrocytic, endothelial, and microglial signals. This signal was resolved in the functional consensus clusters generated using the submodules across functional consensus clusters J, K, and L.
(TIF)

S4 Fig QQ Plots of observed p-values from single-variant association in the ROSMAP cohort. QQ plots of select single-variant association analyses of the DLPFC region that were presented in Fig 3 and S6 Fig show that there is minimal genomic inflation, and consequently, minimal population substructure effects on the analyses. The genomic inflation factor for each QQ plot is also reported. Each QQ plot compares the expected and observed distribution of p-values obtained from the association analysis for a given phenotype.
(TIF)

S5 Fig Subtypes demonstrate no significant enrichment of cognitive or pathological measures. A chi-square test was used to compare distributions of categorical variables and a Student’s t-test was used to compare distributions of quantitative variables between subtypes (α = 0.05 significance level). Braak stages are a measure of neurofibrillary tangles and CERAD scores are a measure of neuritic plaques. Rates of decline in cognitive phenotypes were measured previously by Mukherjee *et al*. for a subset of the ROSMAP cohort.
(TIF)

S6 Fig Manhattan plots of single-variant association of the subtype specificity metric in ROSMAP. Single-variant association of the subtype specificity metric of the two subtypes in the DLPFC region recapitulate multiple loci generally detected at a higher power with submodule eigengenes. Certain loci, such as *MTUS2*, were not detected in previous submodule eigengene associations.
(TIF)

S7 Fig Pathway enrichment analysis for up- and downregulated KEGG pathways among *TMEM106B* rs1990620 haplotype carriers. **(A)** KEGG pathway enrichment analyses of differentially expressed genes among TMEM106B rs1990620 haplotype carriers reveals an upregulation of multiple KEGG pathways associated with neuronal function in deceased patients carrying the protective allele. **(B)** Pathways linked to neuroinflammation and immune function are upregulated in deceased patients carrying the risk haplotype.
(TIF)

S8 Fig Pathway enrichment analysis of differentially expressed genes in subtypes from the ROSMAP cohort. Pathway enrichment analyses of subtypes generated using the DLPFC region data show upregulation of the TREM2/TYROBP pathway in Subtype A and downregulation of the pathway in Subtype B. The KEGG Osteoclast Differentiation pathway and GO Microglial Cell Activation term contain many of the genes associated with the TREM2/TYROBP pathway.
(TIF)

S9 Fig Comparison of differentially expressed genes from the ROSMAP cohort with and without MCI cases. The Venn diagrams depict the results of a sensitivity analysis. The results highlight only marginal differences when including or excluding cases with mild cognitive impairment in the differential expression analysis for the number of: A) Downregulated genes in subtype A. B) Downregulated genes in subtype B. C) Upregulated genes in subtype A. D) Upregulated genes in subtype B.
(TIF)

S10 Fig Pathway enrichment for subtypes from the Mayo and MSBB cohorts. The identified subtypes in the (A) Mayo cohort show a similar pattern in the scaled eigengene expression profiles when compared to the (B) MSBB cohort. Subtypes differ both in the expression of genes linked to inflammatory pathways, such as microglia activation and cellular response to stress, as well as pathways implicated in neuronal function, including synaptic transmission.
(TIF)

S1 Table Summary of cohorts. RNA-Seq and whole genome sequencing data from the Mayo Clinic, the Mount Sinai Brain Bank, and the Rush University's Religious Orders Study and Memory and Aging Project. Six brain regions from these studies were used. The number of RNA-Seq samples and whole genome sequencing data for each tissue are reported.
(XLSX)

S2 Table Summary of cohorts by diagnosis and sex. For each of the six brain regions, possible diagnoses include Late-Onset Alzheimer's Disease (AD), unaffected elderly controls (CONTROL), and other decedents (OTHER). In MSBB and ROSMAP, other decedents were diagnosed with mild cognitive impairment while other decedents in Mayo were diagnosed with either progressive supranuclear palsy (PSP) or pathological aging (PA).
(XLSX)

S3 Table Summary of modules. Modules were generated independently for each tissue region. The number of genes in each module are reported. Twenty-six modules were used in this study.
(XLSX)

S4 Table Summary of submodules. Submodules were generated from existing modules generated for each tissue region. The number of genes in each submodule is reported. Sixty-eight submodules were generated for this study.
(XLSX)

S5 Table GO, KEGG, and Reactome enrichment of submodules. GO and KEGG term enrichment in genes for each submodule was assessed using the clusterProfiler R package for GO and KEGG terms. Reactome term enrichment was similarly assessed using the ReactomePA R package. Enriched terms for each submodule are reported (attached Excel workbook).
(XLSX)

S6 Table Significant SNP associations from TCX region analyses. Significant SNPs that were associated at a genome-wide suggestive level with either a submodule eigengene or the subtype specificity metric are reported. RefSNP IDs are provided if available for the positions, which are aligned to the hg19 human genome build (attached Excel workbook).
(XLSX)

S7 Table Significant SNP associations from PHG region analyses. Significant SNPs that were associated at a genome-wide suggestive level with either a submodule eigengene or the subtype specificity metric are reported. RefSNP IDs are provided if available for the positions, which are aligned to the hg19 human genome build (attached Excel workbook).
(XLSX)

S8 Table Significant SNP associations from FP region analyses. Significant SNPs that were associated at a genome-wide suggestive level with either a submodule eigengene or the subtype specificity metric are reported. RefSNP IDs are provided if available for the positions, which are aligned to the hg19 human genome build (attached Excel workbook).
(XLSX)

S9 Table Significant SNP associations from DLPFC region analyses. Significant SNPs that were associated at a genome-wide suggestive level with either a submodule eigengene or the subtype specificity metric are reported. RefSNP IDs are provided if available for the positions, which are aligned to the hg19 human genome build (attached Excel workbook).
(XLSX)

S10 Table LOAD case subtypes for selected brain regions. Subtypes were generated for the TCX, FP, PHG, and DLPFC regions. 3 clusters were generated for TCX and 2 clusters were generated for the rest. The number of cases in each subtype are reported.
(XLSX)

S11 Table Comparison of clustering algorithms in ROSMAP, MSBB, and Mayo. Assignment of decedents based on different clustering algorithms was compared using a Pearson’s Chi-squared test with Yates’ continuity correction in R. The assignment of decedents was comparable across the four algorithms tested in all three cohorts (attached Excel workbook).
(XLSX)

S12 Table Genome-Wide suggestive SNPs in DLPFC Replicated in TCX, FP, and PHG. SNPs that were found to be genome-wide suggestive in the DLPFC analyses were assessed for replication in the analyses run for the TCX, FP, and PHG regions. A p-value cutoff of 0.05 was used for the TCX, FP, and PHG regions. The analysis that generated the highest p-value for the SNP in each region are reported, along with the p-values from each. (attached Excel workbook).
(XLSX)

S13 Table Genome-Wide suggestive SNPs in DLPFC Replicated in the NHGRI-EBI Catalog. Genome-wide suggestive SNPs from the DLPFC region were assessed for replication in the summary SNPs provided by the NHGRI-EBI catalog (attached Excel workbook).
(XLSX)

S14 Table KEGG and reactome pathway annotations of differentially expressed genes in ROSMAP subtypes. Enrichment of KEGG pathway annotations was assessed for differentially expressed genes between controls and each subtype using the clusterProfiler R package. Enrichment of Reactome pathway annotations was similarly assessed using the ReactomePA R package. Pathways and associated scores are reported (attached Excel workbook).
(XLSX)

## References

[1] Cummings JL. Alzheimer’s Disease. Wood AJJ, editor. N Engl J Med. 2004;351: 56–67. 10.1056/NEJMra040223 15229308

[2] Bertram L, Tanzi RE. Thirty years of Alzheimer’s disease genetics: The implications of systematic meta-analyses. Nat Rev Neurosci. 2008;9: 768–778. 10.1038/nrn2494 18802446

[3] Kilpinen H, Barrett JC. How next-generation sequencing is transforming complex disease genetics. Trends in Genetics. 2013 10.1016/j.tig.2012.10.001 23103023

[4] Risch N, Merikangas K. The future of genetic studies of complex human diseases. Science. 1996 10.1126/science.273.5281.1516 8801636

[5] Jansen IE, Savage JE, Watanabe K, Bryois J, Williams DM, Steinberg S, et al Genome-wide meta-analysis identifies new loci and functional pathways influencing Alzheimer’s disease risk. Nat Genet. 2019;51: 404–413. 10.1038/s41588-018-0311-9 30617256 PMC6836675

[6] Verheijen J, Sleegers K. Understanding Alzheimer Disease at the Interface between Genetics and Transcriptomics. Trends Genet. 2018;34: 434–447. 10.1016/j.tig.2018.02.007 29573818

[7] Mostafavi S, Gaiteri C, Sullivan SE, White CC, Tasaki S, Xu J, et al A molecular network of the aging human brain provides insights into the pathology and cognitive decline of Alzheimer’s disease. Nat Neurosci. 2018;21 10.1038/s41593-018-0154-9 29802388 PMC6599633

[8] Mukherjee S, Mez J, Trittschuh E, Saykin AJ, Gibbons LE, Fardo DW, et al Genetic data and cognitively-defined late-onset Alzheimer’s disease subgroups. Mol Psychiatry. 2018; 1–10. 10.1101/367615 PMC6548676 30514930

[9] Ferreira D, Verhagen C, Hernández-Cabrera JA, Cavallin L, Guo CJ, Ekman U, et al Distinct subtypes of Alzheimer’s disease based on patterns of brain atrophy: longitudinal trajectories and clinical applications. Sci Rep. 2017;7: 1–13. 10.1038/s41598-016-0028-x 28417965 PMC5394684

[10] Langfelder P, Horvath S. Eigengene networks for studying the relationships between co-expression modules. BMC Syst Biol. 2007; 10.1186/1752-0509-1-54 18031580 PMC2267703

[11] Zhang B, Gaiteri C, Bodea LG, Wang Z, McElwee J, Podtelezhnikov AA, et al Integrated systems approach identifies genetic nodes and networks in late-onset Alzheimer’s disease. Cell. 2013; 10.1016/j.cell.2013.03.030 23622250 PMC3677161

[12] De Jager PL, Ma Y, McCabe C, Xu J, Vardarajan BN, Felsky D, et al A multi-omic atlas of the human frontal cortex for aging and Alzheimer’s disease research. Sci Data. 2018;5: 180142 Available: 10.1038/sdata.2018.142 30084846 PMC6080491

[13] Allen M, Carrasquillo MM, Funk C, Heavner BD, Zou F, Younkin CS, et al Human whole genome genotype and transcriptome data for Alzheimer’s and other neurodegenerative diseases. Sci Data. 2016;3: 1–10. 10.1038/sdata.2016.89 27727239 PMC5058336

[14] Wang M, Beckmann ND, Roussos P, Wang E, Zhou X, Wang Q, et al The Mount Sinai cohort of large-scale genomic, transcriptomic and proteomic data in Alzheimer’s disease. Sci Data. 2018;5: 1–16. 10.1038/s41597-018-0002-5 30204156 PMC6132187

[15] Logsdon BA, Perumal TM, Swarup V, Wang M, Funk C, Gaiteri C, et al Meta-analysis of the human brain transcriptome identifies heterogeneity across human AD coexpression modules robust to sample collection and methodological approach. bioRxiv. 2019; 10.7303/syn17114455

[16] Greenfest-Allen E, Cartailler J-P, Magnuson MA, Stoeckert CJ. iterativeWGCNA: iterative refinement to improve module detection from WGCNA co-expression networks. bioRxiv. 2017; 234062. 10.1101/234062

[17] McKenzie AT, Wang M, Hauberg ME, Fullard JF, Kozlenkov A, Keenan A, et al Brain Cell Type Specific Gene Expression and Co-expression Network Architectures. Sci Rep. 2018;8: 1–19. 10.1038/s41598-017-17765-5 29892006 PMC5995803

[18] Kang HM, Sul JH, Service SK, Zaitlen NA, Kong S-Y, Freimer NB, et al Variance component model to account for sample structure in genome-wide association studies. Nat Genet. 2010;42: 348–354. 10.1038/ng.548 20208533 PMC3092069

[19] DeTure MA, Dickson DW. The neuropathological diagnosis of Alzheimer disease. Mol Neurodegener. 2019;14: 1–18. 10.1186/s13024-018-0301-5 31375134 PMC6679484

[20] Charrad M, Ghazzali N, Boiteau V, Niknafs A. NbClust: An R Package for Determining the Relevant Number of Clusters in a Data Set. J Stat Softw. 2014;61: 1–36. 10.18637/jss.v061.i06

[21] White CC, Yang HS, Yu L, Chibnik LB, Dawe RJ, Yang J, et al Identification of genes associated with dissociation of cognitive performance and neuropathological burden: Multistep analysis of genetic, epigenetic, and transcriptional data. PLoS Med. 2017;14: 1–23. 10.1371/journal.pmed.1002287 28441426 PMC5404753

[22] Klein ZA, Takahashi H, Ma M, Stagi M, Zhou M, Lam TKT, et al Loss of TMEM106B Ameliorates Lysosomal and Frontotemporal Dementia-Related Phenotypes in Progranulin-Deficient Mice. Neuron. 2017;95: 281–296.e6. 10.1016/j.neuron.2017.06.026 28728022 PMC5558861

[23] Gallagher MD, Posavi M, Huang P, Unger TL, Berlyand Y, Gruenewald AL, et al A Dementia-Associated Risk Variant near TMEM106B Alters Chromatin Architecture and Gene Expression. Am J Hum Genet. 2017;101: 643–663. 10.1016/j.ajhg.2017.09.004 29056226 PMC5673619

[24] Furney SJ, Simmons A, Breen G, Pedroso I, Lunnon K, Proitsi P, et al Genome-wide association with MRI atrophy measures as a quantitative trait locus for Alzheimer’s disease. Mol Psychiatry. 2011;16: 1130–1138. 10.1038/mp.2010.123 21116278 PMC5980656

[25] Wray NR, Ripke S, Mattheisen M, Trzaskowski M, Byrne EM, Abdellaoui A, et al Genome-wide association analyses identify 44 risk variants and refine the genetic architecture of major depression. Nat Genet. 2018; 10.1038/s41588-018-0090-3 29700475 PMC5934326

[26] Nagel M, Jansen PR, Stringer S, Watanabe K, De Leeuw CA, Bryois J, et al Meta-analysis of genome-wide association studies for neuroticism in 449,484 individuals identifies novel genetic loci and pathways. Nat Genet. 2018; 10.1038/s41588-018-0151-7 29942085

[27] Howard DM, Adams MJ, Clarke TK, Hafferty JD, Gibson J, Shirali M, et al Genome-wide meta-analysis of depression identifies 102 independent variants and highlights the importance of the prefrontal brain regions. Nat Neurosci. 2019; 10.1038/s41593-018-0326-7 30718901 PMC6522363

[28] Nagel M, Watanabe K, Stringer S, Posthuma D, Van Der Sluis S. Item-level analyses reveal genetic heterogeneity in neuroticism. Nat Commun. 2018;9 10.1038/s41467-018-03242-8 29500382 PMC5834468

[29] Luciano M, Hagenaars SP, Davies G, Hill WD, Clarke TK, Shirali M, et al Association analysis in over 329,000 individuals identifies 116 independent variants influencing neuroticism. Nat Genet. 2018; 10.1038/s41588-017-0013-8 29255261 PMC5985926

[30] Baselmans BML, Jansen R, Ip HF, van Dongen J, Abdellaoui A, van de Weijer MP, et al Multivariate genome-wide analyses of the well-being spectrum. Nat Genet. 2019; 10.1038/s41588-018-0320-8 30643256

[31] Hill WD, Weiss A, Liewald DC, Davies G, Porteous DJ, Hayward C, et al Genetic contributions to two special factors of neuroticism are associated with affluence, higher intelligence, better health, and longer life. Mol Psychiatry. 2019; 10.1038/s41380-019-0387-3 30867560 PMC7577854

[32] Van Der Harst P, Verweij N. Identification of 64 novel genetic loci provides an expanded view on the genetic architecture of coronary artery disease. Circ Res. 2018; 10.1161/CIRCRESAHA.117.312086 29212778 PMC5805277

[33] Pottier C, Zhou X, Perkerson RB, Baker M, Jenkins GD, Serie DJ, et al Potential genetic modifiers of disease risk and age at onset in patients with frontotemporal lobar degeneration and GRN mutations: a genome-wide association study. Lancet Neurol. 2018; 10.1016/S1474-4422(18)30126–1 PMC6237181 29724592

[34] Chang D, Nalls MA, Hallgrímsdóttir IB, Hunkapiller J, Brug M van der, Cai F, et al A meta-analysis of genome-wide association studies identifies 17 new Parkinson’s disease risk loci. Nat Genet. 2017; 10.1038/ng.3955 28892059 PMC5812477

[35] MacGregor S, Ong JS, An J, Han X, Zhou T, Siggs OM, et al Genome-wide association study of intraocular pressure uncovers new pathways to glaucoma. Nature Genetics. 2018 10.1038/s41588-018-0176-y 30054594

[36] Gharahkhani P, Burdon KP, Cooke Bailey JN, Hewitt AW, Law MH, Pasquale LR, et al Analysis combining correlated glaucoma traits identifies five new risk loci for open-angle glaucoma. Sci Rep. 2018; 10.1038/s41598-018-20435-9 29449654 PMC5814451

[37] Choquet H, Paylakhi S, Kneeland SC, Thai KK, Hoffmann TJ, Yin J, et al A multiethnic genome-wide association study of primary open-angle glaucoma identifies novel risk loci. Nat Commun. 2018; 10.1038/s41467-018-04555-4 29891935 PMC5995837

[38] Futch HS, Croft CL, Truong VQ, Krause EG, Golde TE. Targeting psychologic stress signaling pathways in Alzheimer’s disease. Mol Neurodegener. 2017;12: 49 10.1186/s13024-017-0190-z 28633663 PMC5479037

[39] Vogl T, Gharibyan AL, Morozova-Roche LA. Pro-Inflammatory S100A8 and S100A9 Proteins: Self-Assembly into Multifunctional Native and Amyloid Complexes. Int J Mol Sci. 2012;13: 2893 10.3390/ijms13032893 22489132 PMC3317694

[40] De Strooper B, Karran E. The Cellular Phase of Alzheimer’s Disease. Cell. 2016;164: 603–615. 10.1016/j.cell.2015.12.056 26871627

[41] Lambert JC, Ibrahim-Verbaas CA, Harold D, Naj AC, Sims R, Bellenguez C, et al Meta-analysis of 74,046 individuals identifies 11 new susceptibility loci for Alzheimer’s disease. Nat Genet. 2013;45: 1452–1458. 10.1038/ng.2802 24162737 PMC3896259

[42] Li Z, Farias FG, Dube U, Del-Aguila JL, Mihindukulasuriya KA, Fernandez MV, et al The TMEM106B rs1990621 protective variant is also associated with increased neuronal proportion. bioRxiv. 2019; 10.1101/583286 PMC6942643 31456032

[43] Schott JM, Crutch SJ, Carrasquillo MM, Uphill J, Shakespeare TJ, Ryan NS, et al Genetic risk factors for the posterior cortical atrophy variant of Alzheimer’s disease. Alzheimer’s Dement. 2016;12: 862–871. 10.1016/j.jalz.2016.01.010 26993346 PMC4982482

[44] Kichaev G, Bhatia G, Loh P-R, Gazal S, Burch K, Freund MK, et al Leveraging Polygenic Functional Enrichment to Improve GWAS Power. Am J Hum Genet. 2019;104: 65–75. 10.1016/j.ajhg.2018.11.008 30595370 PMC6323418

[45] Chibnik LB, White CC, Mukherjee S, Raj T, Yu L, Larson EB, et al Susceptibility to neurofibrillary tangles: role of the PTPRD locus and limited pleiotropy with other neuropathologies. Mol Psychiatry. 2018;23: 1521–1529. 10.1038/mp.2017.20 28322283 PMC5608624

[46] Rousseeuw PJ. Silhouettes: a graphical aid to the interpretation and validation of cluster analysis. J Comput Appl Math. 1986;20: 53–65. 10.1177/003754977702900403

[47] Clifford H, Wessely F, Pendurthi S, Emes RD. Comparison of clustering methods for investigation of genome-wide methylation array data. Front Genet. 2011;2: 1–11. 10.3389/fgene.2011.00001 22303382 PMC3268382

[48] Braak H, Thal DR, Ghebremedhin E, Del Tredici K. Stages of the Pathologic Process in Alzheimer Disease. J Neuropathol Exp Neurol. 2011;70: 960–969. 10.1097/NEN.0b013e318232a379 22002422

[49] Wilson RS, Arnold SE, Schneider JA, Li Y, Bennett DA. Chronic Distress, Age-Related Neuropathology, and Late-Life Dementia. Psychosom Med. 2007;69 Available: https://journals.lww.com/psychosomaticmedicine/Fulltext/2007/01000/Chronic_Distress,_Age_Related_Neuropathology,_and.9.aspx 10.1097/01.psy.0000250264.25017.21 17244848

[50] Mukherjee S, Mez J, Trittschuh EH, Saykin AJ, Gibbons LE, Fardo DW, et al Genetic data and cognitively defined late-onset Alzheimer’s disease subgroups. Mol Psychiatry. 2018; 10.1038/s41380-018-0298-8 30514930 PMC6548676

[51] Ritchie ME, Phipson B, Wu D, Hu Y, Law CW, Shi W, et al limma powers differential expression analyses for RNA-sequencing and microarray studies. Nucleic Acids Res. 2015;43: e47–e47. 10.1093/nar/gkv007 25605792 PMC4402510

[52] Yu G, Wang L-G, Han Y, He Q-Y. clusterProfiler: an R Package for Comparing Biological Themes Among Gene Clusters. Omi A J Integr Biol. 2012;16: 284–287. 10.1089/omi.2011.0118 22455463 PMC3339379

[53] Buniello A, Macarthur JAL, Cerezo M, Harris LW, Hayhurst J, Malangone C, et al The NHGRI-EBI GWAS Catalog of published genome-wide association studies, targeted arrays and summary statistics 2019. Nucleic Acids Res. 2019;47: D1005–D1012. 10.1093/nar/gky1120 30445434 PMC6323933

[54] Shannon P, Markiel A, Ozier O, Baliga NS, Wang JT, Ramage D, et al Cytoscape: A Software Environment for Integrated Models. Genome Res. 2003;13: 2498–2504. 10.1101/gr.1239303 14597658 PMC403769

