# Supplementary figures and images for: Transcriptomic stratification of late-onset Alzheimer's cases reveals novel genetic modifiers of disease pathology
Source: PLoS Genet. 2020 Jun 3;16(6):e1008775. doi: 10.1371/journal.pgen.1008775 (PMC7295244; doi:10.1371/journal.pgen.1008775)

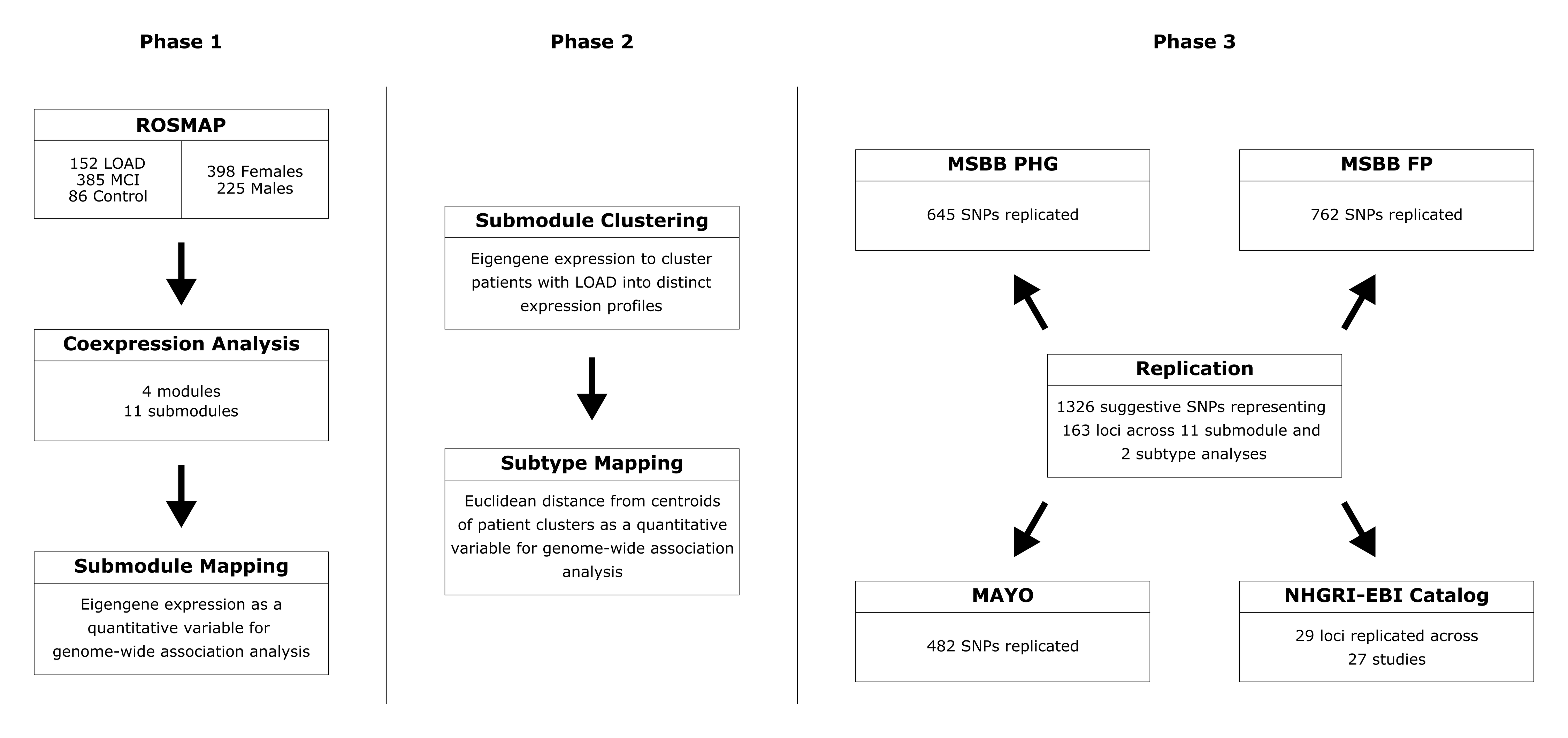

Supplement: S1 Fig — Phase 1 involved the co-expression analysis of ROSMAP and other cohorts to generate submodules representing biological processes involved in Alzheimer’s pathology. Eigengene expression from the submodules were used to perform single-variant association and identify loci that act as putative genetic drivers of these biological pathways. Phase 2 involved the clustering of LOAD cases in ROSMAP and other cohorts based on an agnostic clustering method. Subtypes were mapped using single-variant association to identify loci that may explain the heterogeneity observed in LOAD cases. Phase 3 involved the replication of genome-wide suggestive or genome-wide significant SNPs from the ROSMAP cohort in other tissue regions and previous studies. SNPs were replicated in three other tissue regions (PHG, FP, TCX) and in 27 studies from the NHGRI-EBI catalog. (TIF) [file pgen.1008775.s001.tif]

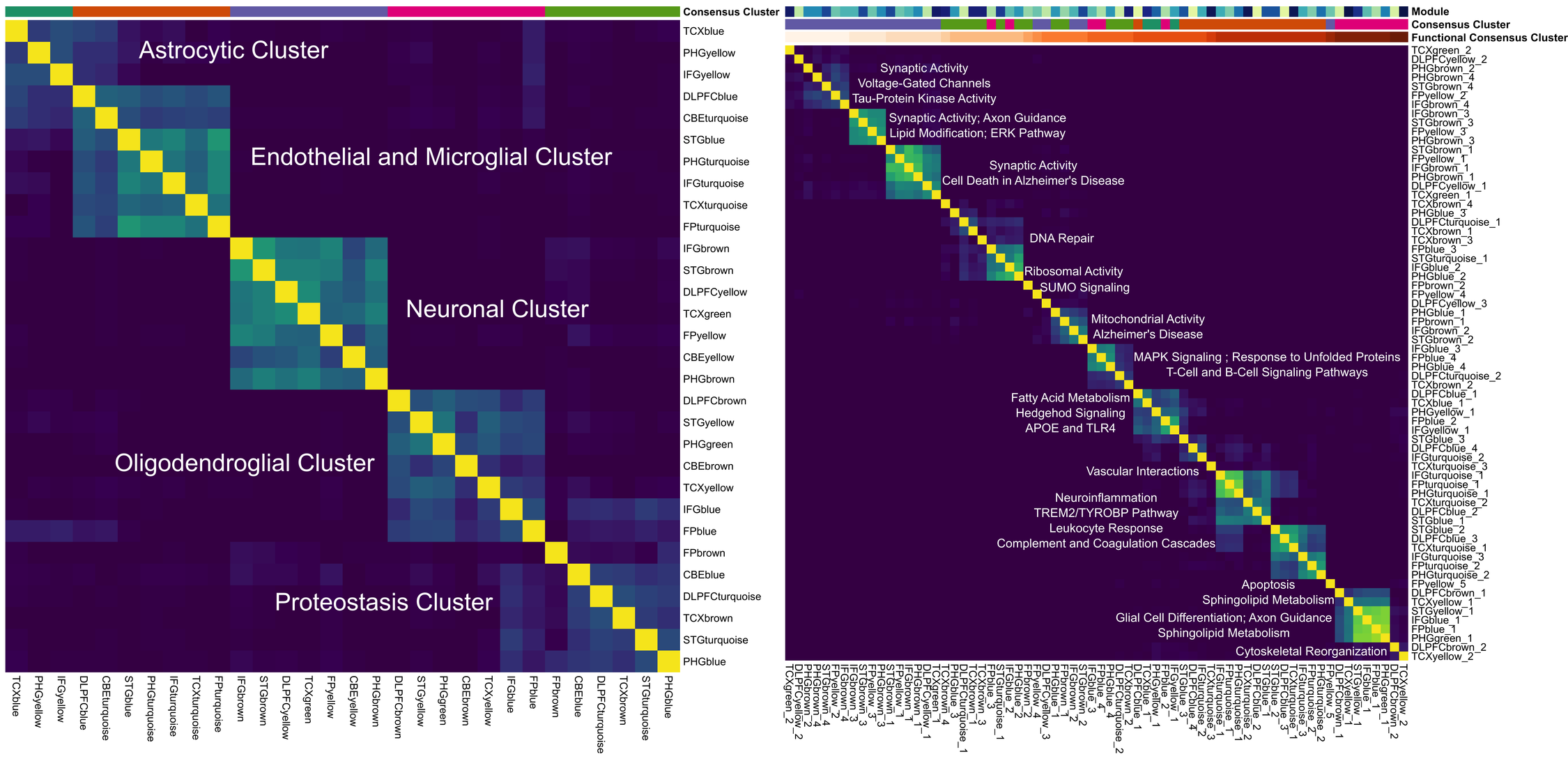

Supplement: S2 Fig — (A) A previous study by Logsdon et al. reported 5 consensus clusters across 7 tissue regions based on the modules generated for each tissue region. A Jaccard matrix heatmap is used to visualize the overlap of genes in each module between tissue regions and cohorts. (B) Submodules were divided into 15 functional clusters based on hierarchical clustering that demonstrated specificity for certain biological pathways. These functional clusters formed independently of module of origin and tissue of origin. (TIF) [file pgen.1008775.s002.tif]

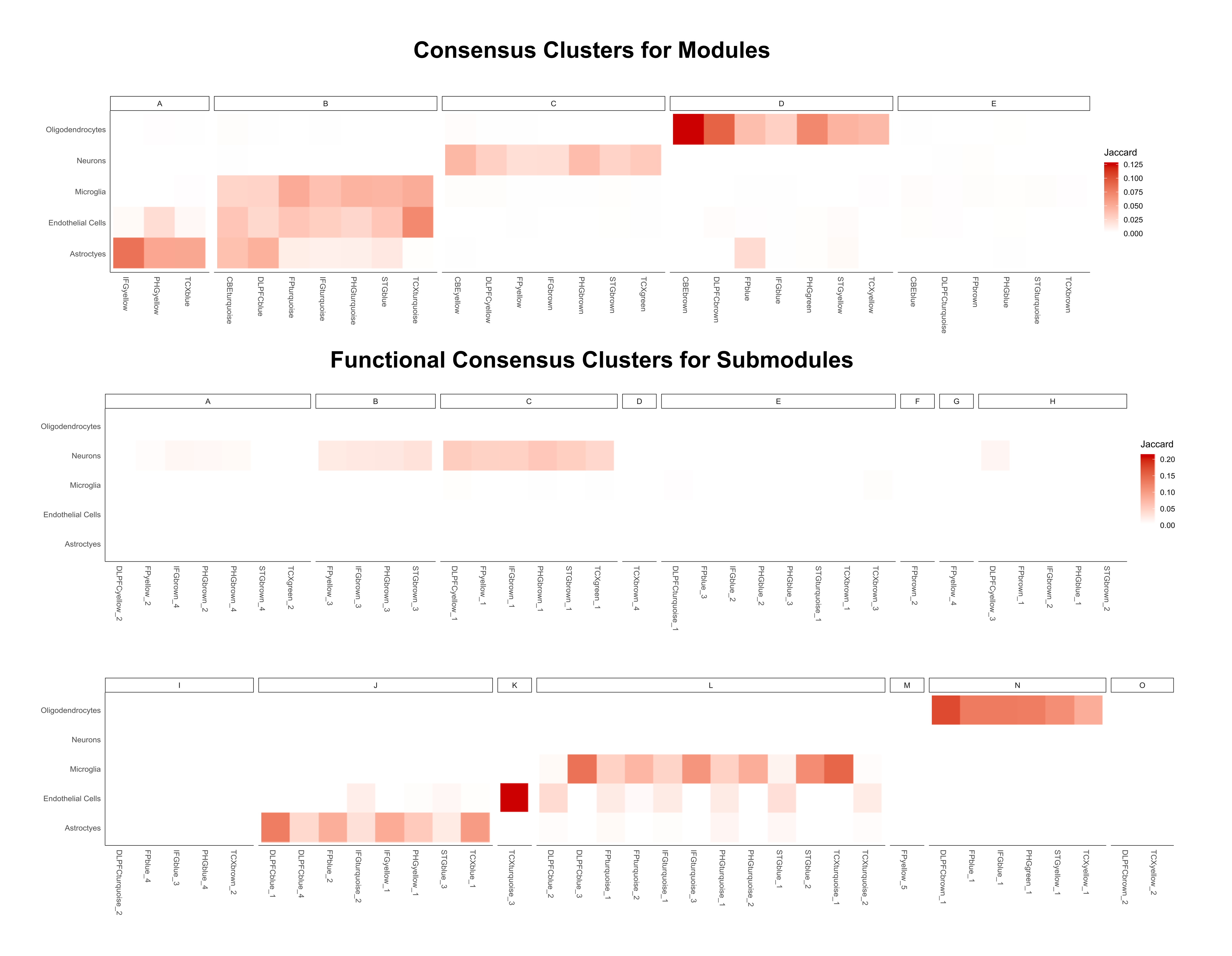

Supplement: S3 Fig — Brain tissue cell-type specific markers reported previously by McKenzie et al. were used to assess the cell-type specificity of modules and submodules. Consensus clusters B broadly captured astrocytic, endothelial, and microglial signals. This signal was resolved in the functional consensus clusters generated using the submodules across functional consensus clusters J, K, and L. (TIF) [file pgen.1008775.s003.tif]

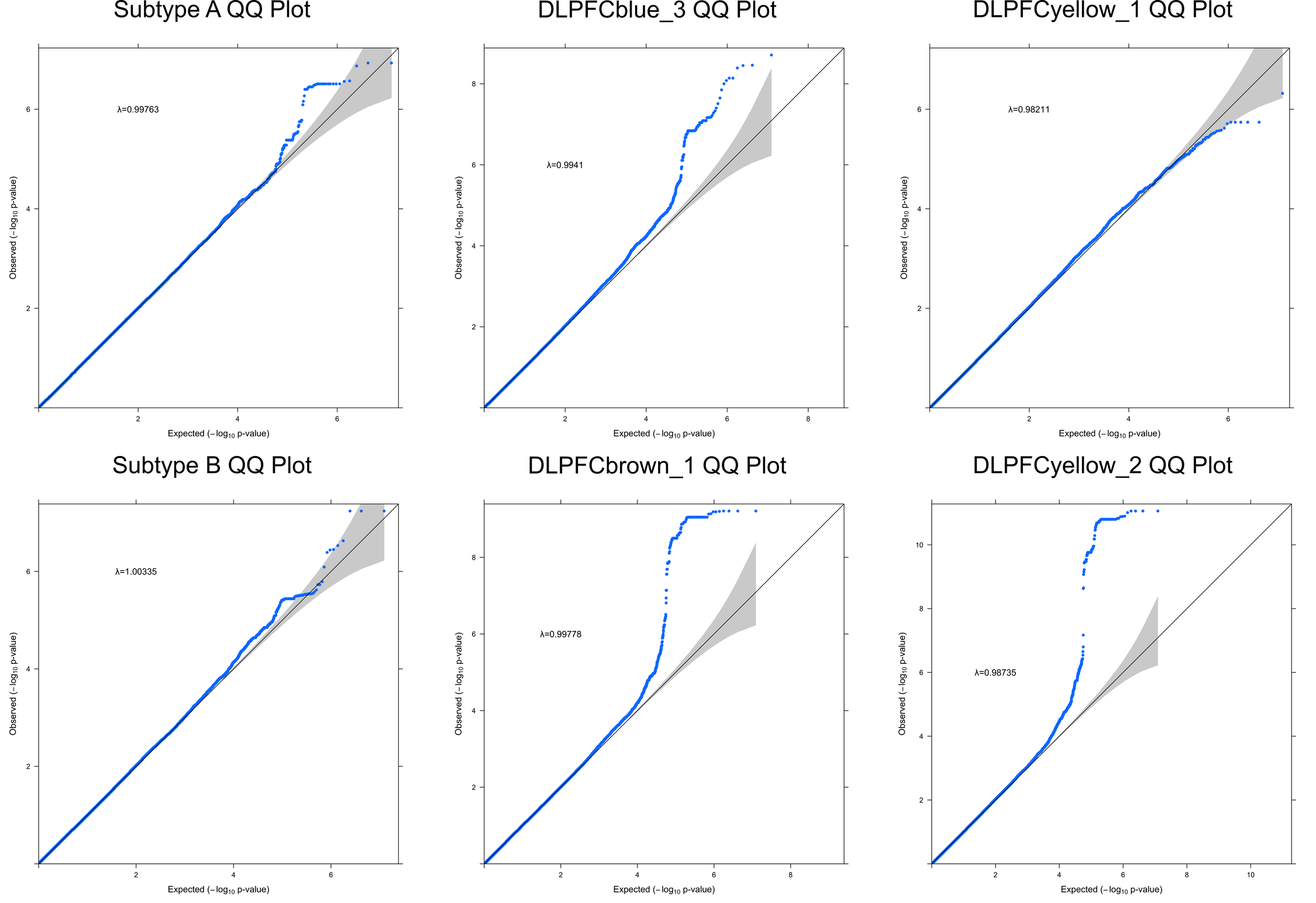

Supplement: S4 Fig — QQ plots of select single-variant association analyses of the DLPFC region that were presented in Fig 3 and S6 Fig show that there is minimal genomic inflation, and consequently, minimal population substructure effects on the analyses. The genomic inflation factor for each QQ plot is also reported. Each QQ plot compares the expected and observed distribution of p-values obtained from the association analysis for a given phenotype. (TIF) [file pgen.1008775.s004.tif]

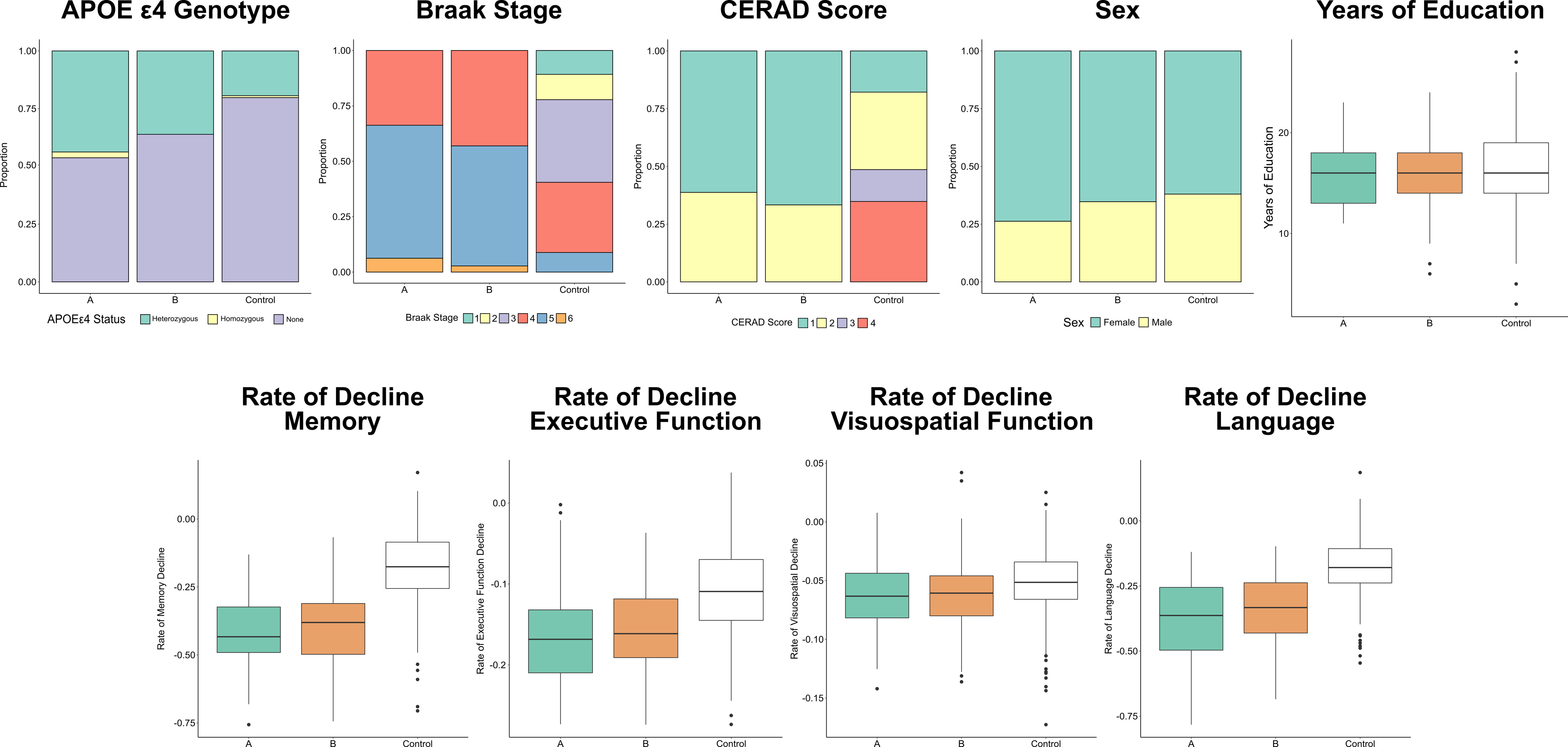

Supplement: S5 Fig — A chi-square test was used to compare distributions of categorical variables and a Student’s t-test was used to compare distributions of quantitative variables between subtypes (α = 0.05 significance level). Braak stages are a measure of neurofibrillary tangles and CERAD scores are a measure of neuritic plaques. Rates of decline in cognitive phenotypes were measured previously by Mukherjee et al. for a subset of the ROSMAP cohort. (TIF) [file pgen.1008775.s005.tif]

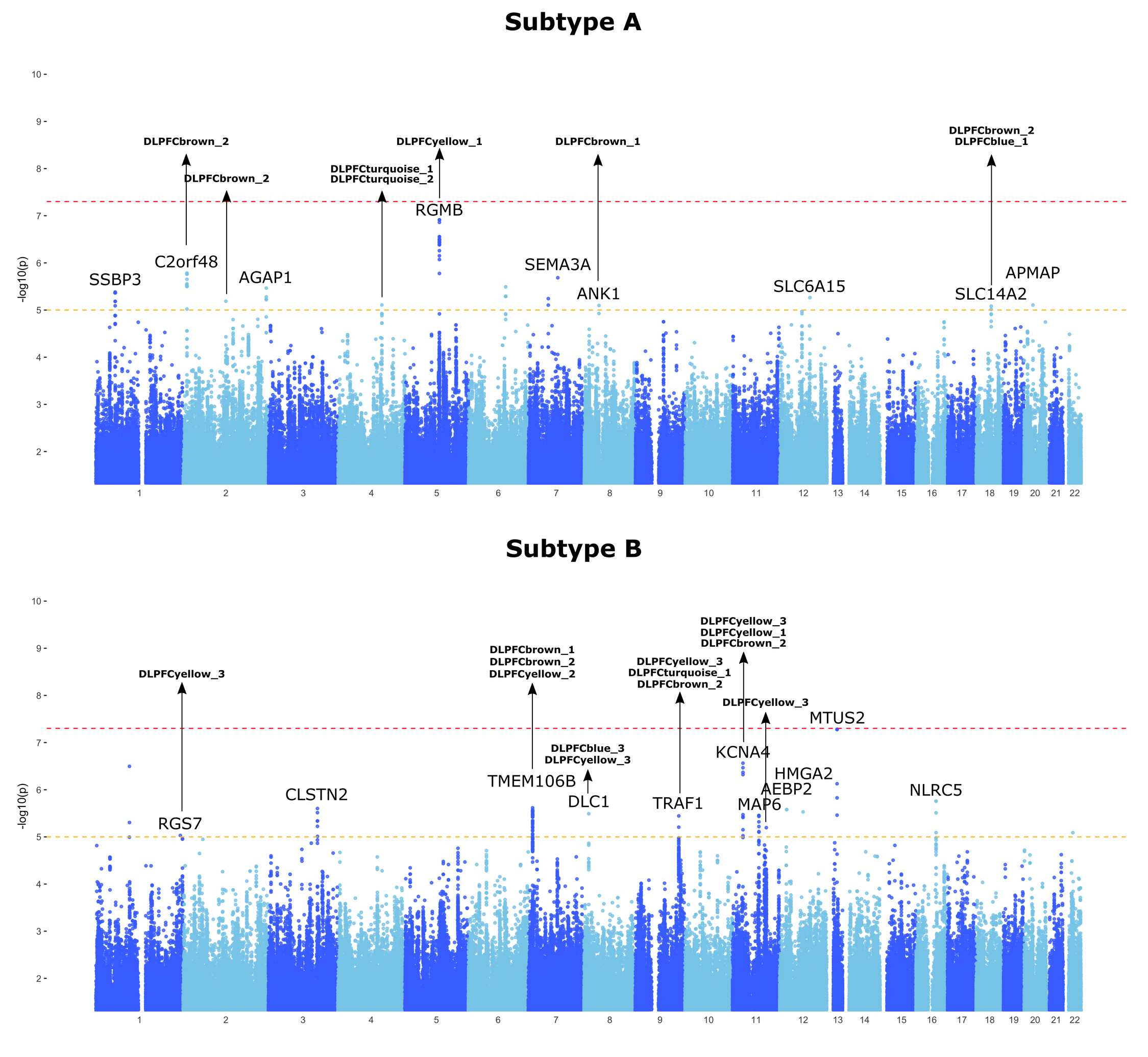

Supplement: S6 Fig — Single-variant association of the subtype specificity metric of the two subtypes in the DLPFC region recapitulate multiple loci generally detected at a higher power with submodule eigengenes. Certain loci, such as MTUS2, were not detected in previous submodule eigengene associations. (TIF) [file pgen.1008775.s006.tif]

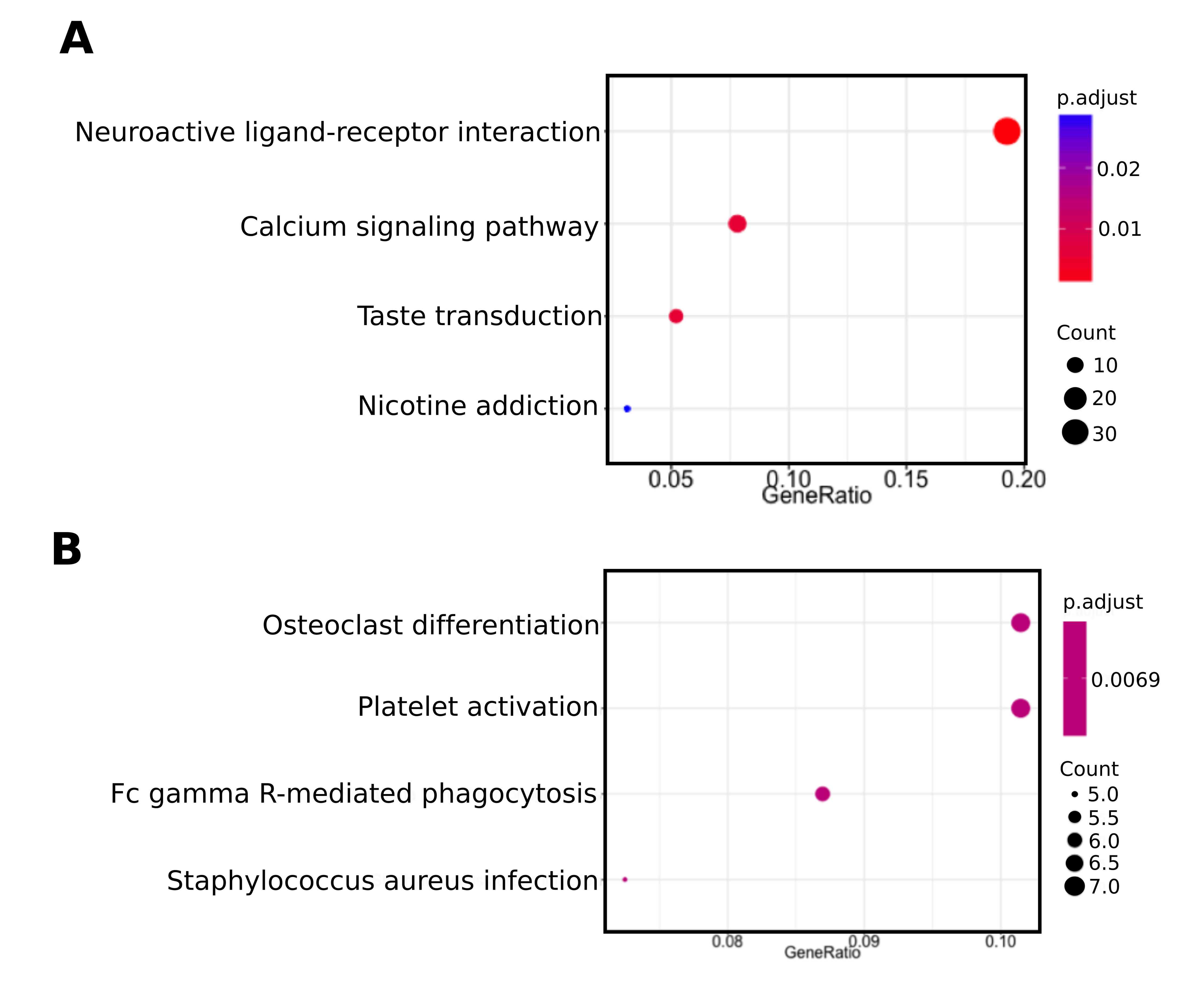

Supplement: S7 Fig — (A) KEGG pathway enrichment analyses of differentially expressed genes among TMEM106B rs1990620 haplotype carriers reveals an upregulation of multiple KEGG pathways associated with neuronal function in deceased patients carrying the protective allele. (B) Pathways linked to neuroinflammation and immune function are upregulated in deceased patients carrying the risk haplotype. (TIF) [file pgen.1008775.s007.tif]

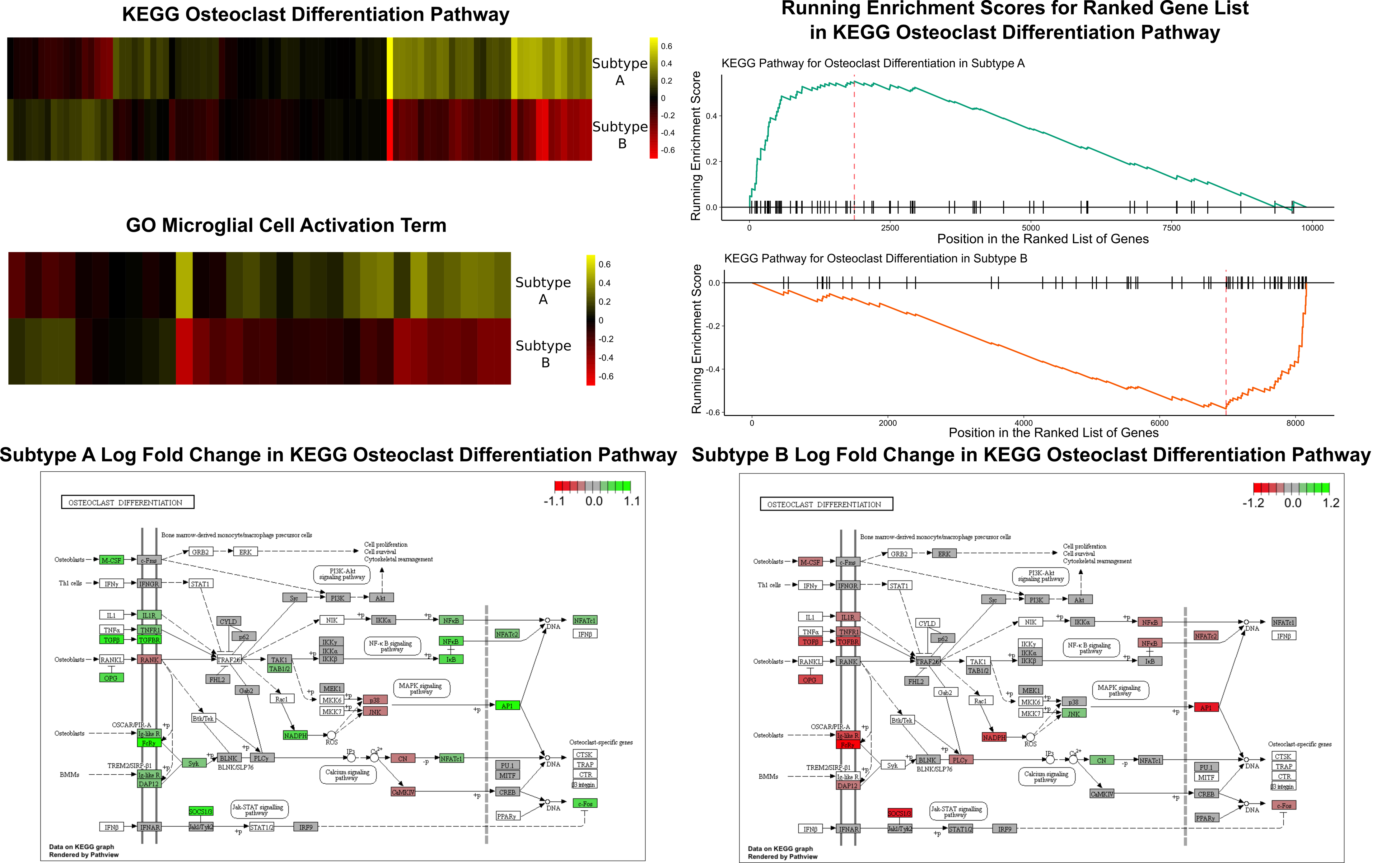

Supplement: S8 Fig — Pathway enrichment analyses of subtypes generated using the DLPFC region data show upregulation of the TREM2/TYROBP pathway in Subtype A and downregulation of the pathway in Subtype B. The KEGG Osteoclast Differentiation pathway and GO Microglial Cell Activation term contain many of the genes associated with the TREM2/TYROBP pathway. (TIF) [file pgen.1008775.s008.tif]

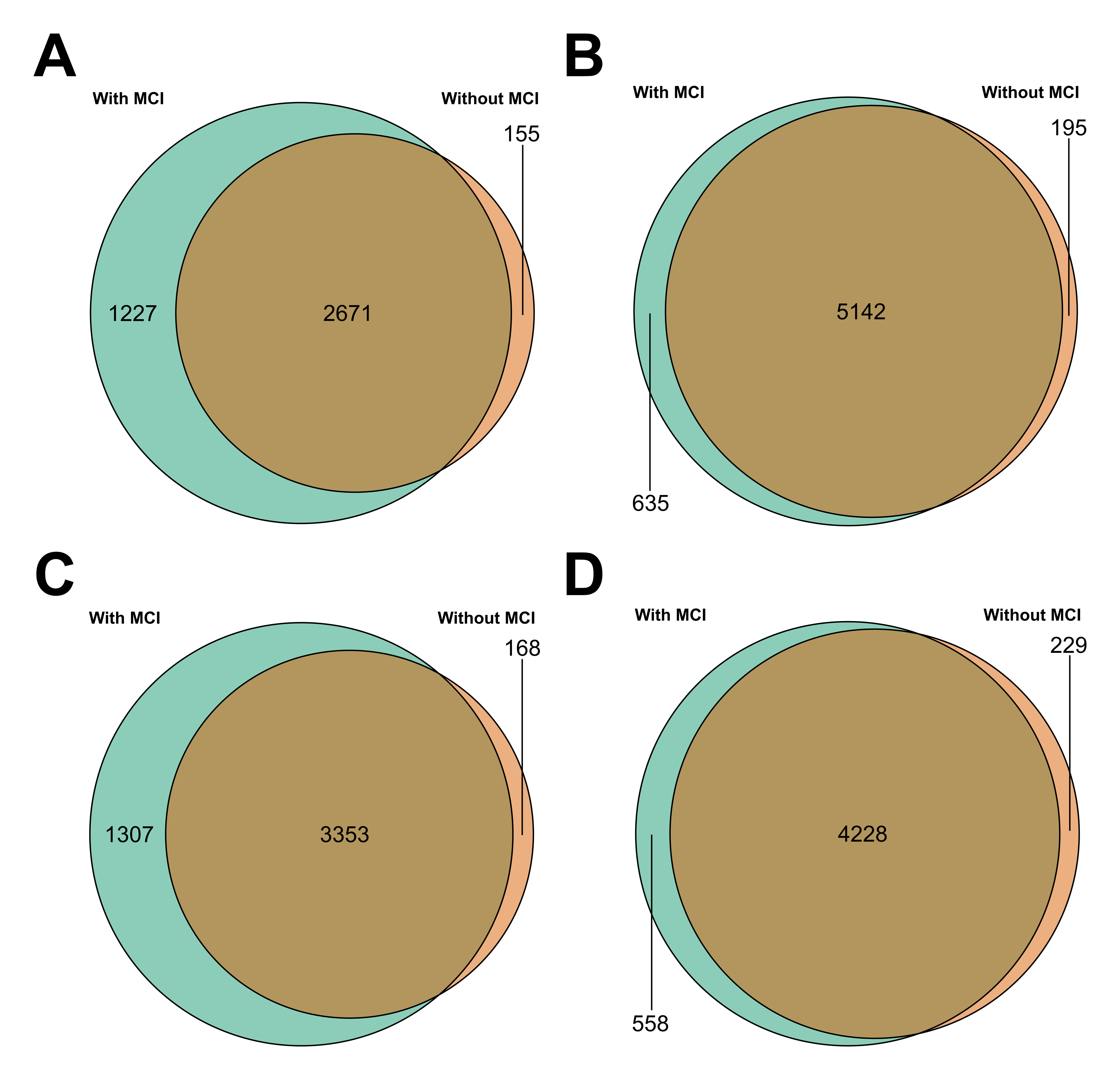

Supplement: S9 Fig — The Venn diagrams depict the results of a sensitivity analysis. The results highlight only marginal differences when including or excluding cases with mild cognitive impairment in the differential expression analysis for the number of: A) Downregulated genes in subtype A. B) Downregulated genes in subtype B. C) Upregulated genes in subtype A. D) Upregulated genes in subtype B. (TIF) [file pgen.1008775.s009.tif]

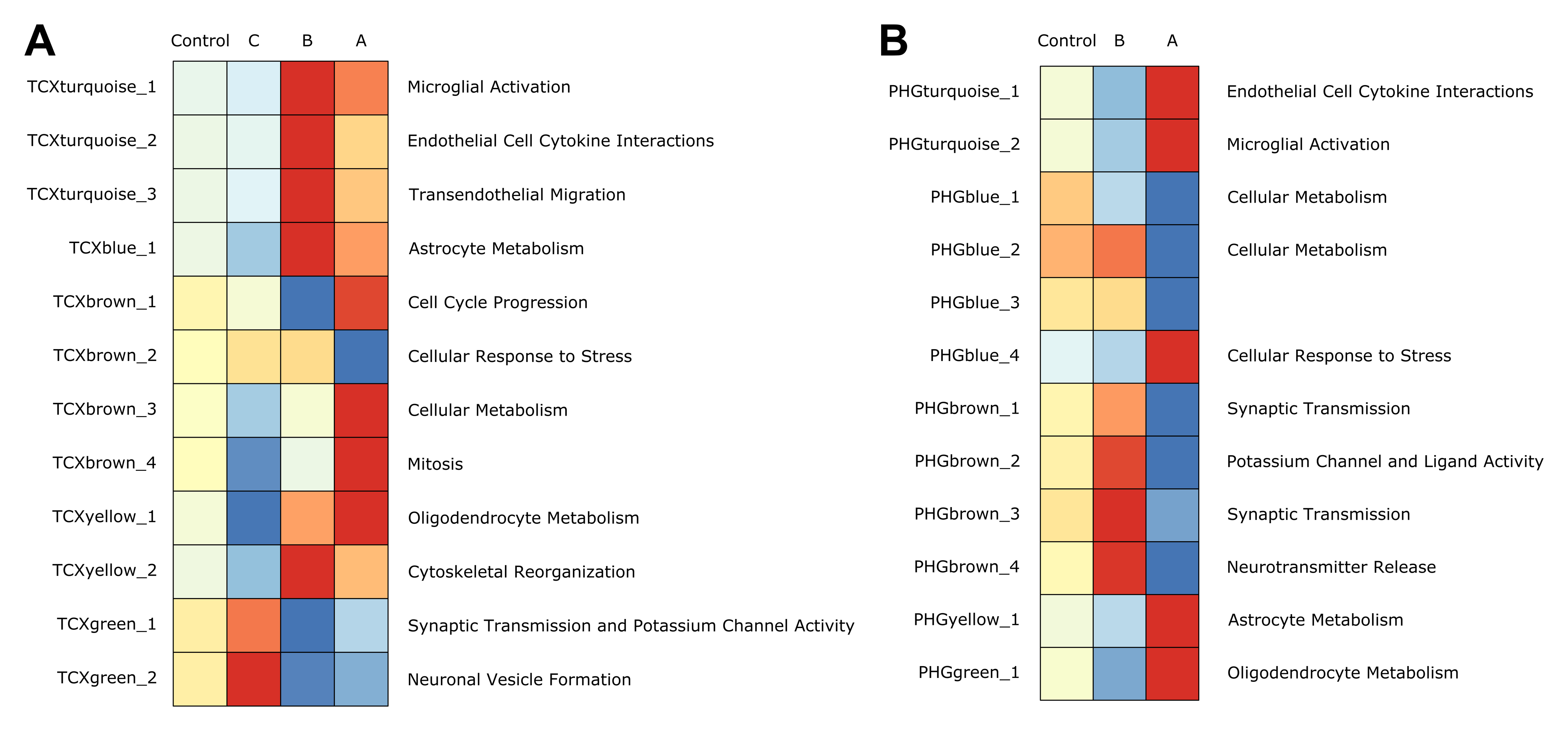

Supplement: S10 Fig — The identified subtypes in the (A) Mayo cohort show a similar pattern in the scaled eigengene expression profiles when compared to the (B) MSBB cohort. Subtypes differ both in the expression of genes linked to inflammatory pathways, such as microglia activation and cellular response to stress, as well as pathways implicated in neuronal function, including synaptic transmission. (TIF) [file pgen.1008775.s010.tif]
